# TNF receptor agonists induce distinct receptor clusters to mediate differential agonistic activity

**DOI:** 10.1038/s42003-021-02309-5

**Published:** 2021-06-23

**Authors:** Xiaojie Yu, Sonya James, James H. Felce, Blanka Kellermayer, David A. Johnston, H. T. Claude Chan, Christine A. Penfold, Jinny Kim, Tatyana Inzhelevskaya, C. Ian Mockridge, Yasunori Watanabe, Max Crispin, Ruth R. French, Patrick J. Duriez, Leon R. Douglas, Martin J. Glennie, Mark S. Cragg

**Affiliations:** 1 Antibody and Vaccine Group, School of Cancer Sciences, University of Southampton Faculty of Medicine, Southampton, UK; 2 ONI UK, Linacre House, Banbury Road, Oxford, UK; 3 Biomedical Imaging Unit, University of Southampton Faculty of Medicine, Southampton, UK; 4 School of Biological Sciences, University of Southampton, Southampton, UK; 5 CRUK Protein Core Facility, University of Southampton Faculty of Medicine, Southampton, UK; 6 Institute for Life Sciences, University of Southampton, Southampton, UK

**Keywords:** Imaging the immune system, Biological fluorescence

## Abstract

Monoclonal antibodies (mAb) and natural ligands targeting costimulatory tumor necrosis factor receptors (TNFR) exhibit a wide range of agonistic activities and antitumor responses. The mechanisms underlying these differential agonistic activities remain poorly understood. Here, we employ a panel of experimental and clinically-relevant molecules targeting human CD40, 4-1BB and OX40 to examine this issue. Confocal and STORM microscopy reveal that strongly agonistic reagents induce clusters characterized by small area and high receptor density. Using antibody pairs differing only in isotype we show that hIgG2 confers significantly more receptor clustering than hIgG1 across all three receptors, explaining its greater agonistic activity, with receptor clustering shielding the receptor-agonist complex from further molecular access. Nevertheless, discrete receptor clustering patterns are observed with different hIgG2 mAb, with a unique rod-shaped assembly observed with the most agonistic mAb. These findings dispel the notion that larger receptor clusters elicit greater agonism, and instead point to receptor density and subsequent super-structure as key determinants.

## Abstract (web summary)

Yu et al examined a panel of both experimental and clinically-relevant TNF agonists in order to advance our understanding of the mechanisms underlying their varying activities and anti-tumor responses. They demonstrated that agonists with greater activity induced higher density receptor clustering and specific super-structures as opposed to simply larger receptor clusters.

## Introduction

Costimulatory tumour necrosis factor receptors (TNFRs) play important roles in immune activation and represent promising targets for next-generation cancer immunotherapeutics^1–3^. Selective TNFR activation has been shown to augment antitumor immunity and confer robust therapeutic benefits in animal models^1^ with TNFR-targeting mAbs and recombinant ligands showing promising antitumor activities in early phase trials^1,4,5^, although they remain behind checkpoint-blocking reagents in their development, likely due to a poorer understanding of how the activity is achieved and their multiple potential mechanisms of action^6,7^. These clinical candidates display a range of agonistic activities resulting from differential TNFR activation, with their level of agonism correlated positively with their pharmacodynamic properties in clinical trials^8–13^.

TNFR activation follows the paradigm of ligand-induced receptor oligomerization^4,14^. TNFR are type I transmembrane proteins characterised by the presence of 3–4 extracellular cysteine-rich domains (CRD) and signal via cytoplasmic association with TRAF, FADD or TRADD adaptor proteins^4,15^. Endogenous TNFR ligands are type II transmembrane proteins sharing the common extracellular TNF homology domain that drives non-covalent trimerization^4^. These membrane-bound ligands can undergo proteolysis to form soluble trimers detectable in circulation^2^. With a few exceptions^16,17^, most crystal structures of TNFR-ligand complexes infer a conserved mode of interaction underpinning activation whereby a trimeric ligand engages three receptor monomers, forming the basic unit of TNFR signalling^10,18–23^.

In contrast to their natural ligands, the oligomeric state of TNFRs remains unclear, with growing evidence suggesting that at least some TNFRs exist as pre-assembled oligomers on the cell surface. For example, chemical crosslinking of the cell surface indicates that TNFR1, TNFR2, Fas, TRAILR2 and CD40 can self-associate into non-covalent dimers or trimers, mediated by the pre-ligand assembly domain (PLAD) within CRD1 via homotypic interaction^24–28^. Super-resolution microscopy quantified TNFR1 to comprise 66% monomer and 34% dimer on the cell surface, shifting to 13% monomer, 64% trimer and 23% higher-order oligomer upon soluble TNF-α binding^29^. Moreover, a dysfunctional PLAD restricted TNFR1 to monomers even in the presence of TNF-α^29^. Further biophysical characterisations of recombinant PLADs of TNFR1 and TNFR2 detected some specific, albeit weak, PLAD–PLAD interactions^30^.

While the significance of the PLAD module in each TNFR continues to be unravelled, the concept of PLAD provides the mechanistic basis for a two-step model of ligand-induced TNFR activation, which proposes that the initial formation of the trimeric receptor-ligand complex (step one) is followed by higher-order oligomerization via PLAD interactions between adjacent complexes (step two)^4^. TNFR ligands typically engage TNFRs via a conserved interface within CRD2 and CRD3; however, rules dictating agonistic anti-TNFR mAb activity are more complex^4,14^. We previously demonstrated that a complex interplay between epitope and isotype determines anti-TNFR mAb agonism, with the human IgG2 (hIgG2) isotype able to impart superior agonistic activity and convert CD40 antagonism to agonism^8,9,31^.

Although the two-step model and PLAD concept offer a molecular basis for TNFR activation, our understanding of the relationship between receptor clustering and agonistic activity has remained largely binary, unable to account for the range of agonism displayed by the variety of reagents so far explored in the clinic^11–13,32^. At least for some TNFR targets, a lack of agonism has been associated with subsequent clinical failure^11,12^, while excessive agonism has been linked to toxicity-related complications^13,32^. A better understanding of the molecular features that underlie differential TNFR agonism is critical for the generation of agonists with the desired level of agonistic activity needed to elicit therapeutic benefit. Here we employed a panel of clinically relevant mAbs and recombinant natural ligands targeting three different TNFRs (CD40, 4-1BB and OX40) to investigate features of receptor clustering that underlie differential agonism. We found that different agonists triggered distinct patterns of receptor clustering, with natural ligands and strong agonists inducing small clusters with higher receptor density. Receptors enclosed within these clusters were refractory to further molecular access. Moreover, a higher-order structure, manifested as distinct rod-shaped clusters, was uniquely observed for our most potent CD40 super-agonist. This work reveals that diverse TNFR agonism is achieved through distinct receptor clustering, which underlies the fundamental mechanism of TNFR activation.

## Results

### TNFR agonists induce receptor clustering

**Fig. 1:**
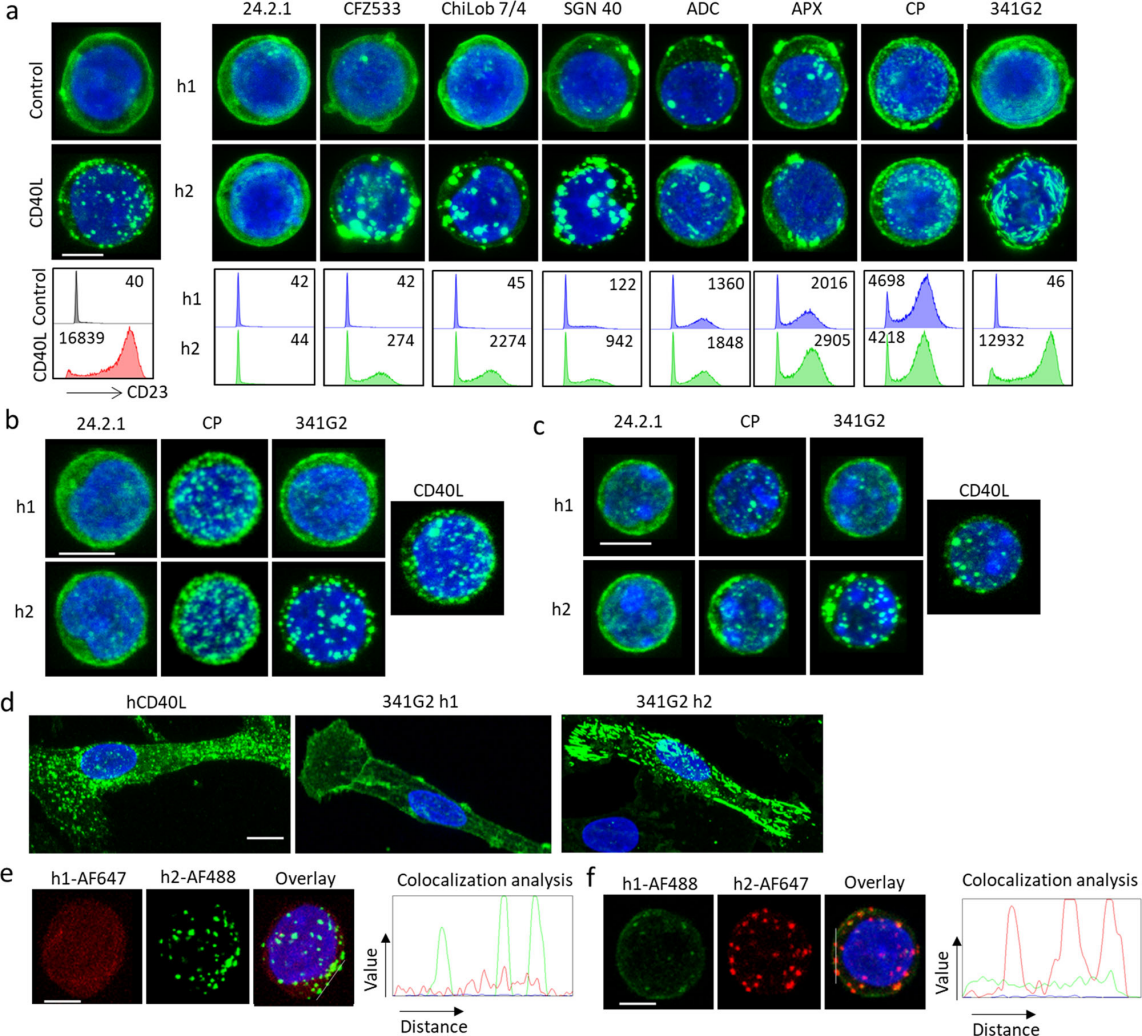
CD40 agonists induce differential CD40 clustering. **a** Upper panels: Jurkat-hCD40ECD-GFP cells were treated with 10 µg/mL anti-CD40 mAbs of either hIgG1 (h1) or hIgG2 (h2) isotype as indicated for 1 h, and then fixed with methanol, nucleus stained with DAPI and imaged using a Leica SP8 confocal microscope. Lower panels: B cells isolated from hCD40Tg/FcγR null mice were incubated with various anti-CD40 mAbs at 10 µg/mL as indicated for 3 days and then stained for surface expression of CD23 and analysed by flow cytometry, CD23 MFI indicated within histogram was quantified by Flowjo Software. **b** Normal human B cells isolated from healthy donor PBMC, or **c** B cells isolated from hCD40Tg mice were stained with AF488-labelled anti-CD40 mAbs as indicated for 1 h and then fixed with methanol, nucleus stained with DAPI and imaged using a Leica SP8 confocal microscope. **d** Human monocyte-derived DCs were stained with 10 µg/mL AF488-labelled hCD40L, 341G2 h1 or 341G2 h2 for 1 h and then fixed with methanol, nucleus stained with DAPI and imaged using a Leica SP8 confocal microscope. **e** Jurkat-hCD40ECD cells were co-stained with 341G2 h1-AF647 and 341G2 h2-AF488 or **f** co-stained with 341G2 h1-AF488 and 341G2 h2-AF647 for 1 h, and then fixed with methanol, nucleus stained with DAPI and imaged using a Leica SP8 confocal microscope; co-localisation analysis performed using ImageJ. All images are representative of at least ten images from at least two independent experiments. Scale bar, 4 µm.

Clinically relevant TNFR agonists, comprising mAbs and recombinant natural ligands, exhibit a range of antitumor activities in cancer patients. To dissect the molecular mechanisms underlying such differential TNFR activation, we first investigated CD40 as a paradigm and explored receptor clustering on Jurkat cells expressing human CD40 extracellular domain (ECD) coupled to GFP. Cells lacking the CD40 intracellular domain were employed initially to reduce complexity arising from potential signalling activities within the cell. We assessed a panel of clinically relevant CD40 agonists possessing a range of agonistic activities^8,31^, spanning non-agonistic (24.2.1) to super-agonist (341G2 hIgG2) mAb, as agonistic as CD40L. 341G2 hIgG2 is 4 times more potent than CP870,893 the most agonistic anti-CD40 mAb so far examined in the clinic^31^ (Supplementary Fig. 1). We previously demonstrated that the wild type hIgG2 isotype typically imparts stronger agonistic potential compared to hIgG1^8,9^ and so examined mAb in both isotypes. Confocal microscopy showed that these various reagents induced distinct patterns of receptor clusters varying in size and shape (Fig. 1a). Overall, clustering in these Jurkat cells directly correlated with agonism as measured by CD23 upregulation on human CD40 transgenic (hCD40Tg) B cells lacking FcγRs (Fig. 1a, histograms). This association between receptor clustering and agonism was evident for mAb that lacked activity in any isotype (24.2.1), those that were active following isotype switching to hIgG2 (exemplified by 341G2) and those active in both isotypes including the previously reported “super-agonist” CP870,893 (Fig. 1a). Nonetheless, as before we observed the wild type hIgG2 isotype conferred greater activity and clustering for the majority of mAb. Importantly, such receptor clustering was recapitulated in Jurkat cells expressing full-length CD40 (Supplementary Fig. 2a) and in a heterologous CHO-k1 cell system expressing CD40ECD-GFP (Supplementary Fig. 2b); indicating that the degree of clustering reflects inherent cross-linking/clustering properties of the mAb, rather than a particular cell system context. F(ab’)_2_ but not Fab fragments of 341G2 hIgG2 and CP870,893 hIgG2 both induced similar receptor clustering as the full-length IgG (Supplementary Fig. 2c), consistent with our previous findings that hIgG2-mediated agonism requires bivalency but is Fc-independent^8,9,31^.

To confirm these observations in physiologically relevant cell populations, we stained CD40-expressing immune cells with labelled anti-CD40 mAbs or CD40L. Consistent with the data above, the same differential receptor clustering was recapitulated on Ramos malignant B cells (Supplementary Fig. 2d,) normal human B cells (Fig. 1b), murine hCD40Tg B cells (Fig. 1c), and human monocyte-derived dendritic cells (DCs) (Fig. 1d). To further examine the profound ability of the wild type hIgG2 isotype to elicit receptor clustering, we co-stained Jurkat CD40ECD-expressing cells with differentially labelled hIgG1 and hIgG2 variants of 341G2 and found that the hIgG2 isotype, regardless of the fluorescent label, induced distinct clusters while hIgG1 exhibited more dispersed staining throughout the same cell surface (Fig. 1e, f and Supplementary Fig. 2e), demonstrating that hIgG2-mediated clustering does not relate to any unanticipated property of the fluorochrome and that it does not elicit clustering of all available surface CD40 molecules.

We previously documented that hyper-crosslinking provided by secondary antibodies or accessory cells over-expressing FcγR can obviate the inherent agonistic potential of anti-mouse or anti-human CD40 mAb to deliver powerful agonism^8,33^. To explore this observation with respect to receptor clustering, we co-incubated Jurkat CD40ECD-GFP cells with CHO-k1 cells over-expressing human FcγR1A, FcγR2A or FcγR2B. All three cell-types, but not native CHO-k1 cells, conferred extensive receptor redistribution to the cell-cell interface, concurrent with maximal NFκB activation with all hIgG1 mAb (Supplementary Fig. 3) irrespective of inherent activity, including with the native antagonist 24.2.1.

As endogenous CD40 activation occurs through engagement with CD40L on activated CD4 T cells, we also examined receptor clustering after co-culturing activated CD40L-expressing human CD4+ T cells (Supplementary Fig. 4a) with various CD40-expressing cells including Jurkat CD40ECD-GFP cells (Supplementary Fig. 4b), monocyte-derived DCs (Supplementary Fig. 4c) and normal human B cells (Supplementary Fig. 4d), and observed significant clustering at the cell-cell interface akin to that induced by FcγR-expressing CHO-k1 cells (Supplementary Fig. 3a). Moreover, such clusters were inhibited by the antagonist 24.2.1 (which binds within the CD40L binding site) but not ChiLob 7/4 (which does not) (Supplementary Fig. 4b)^8^.

**Fig. 2:**
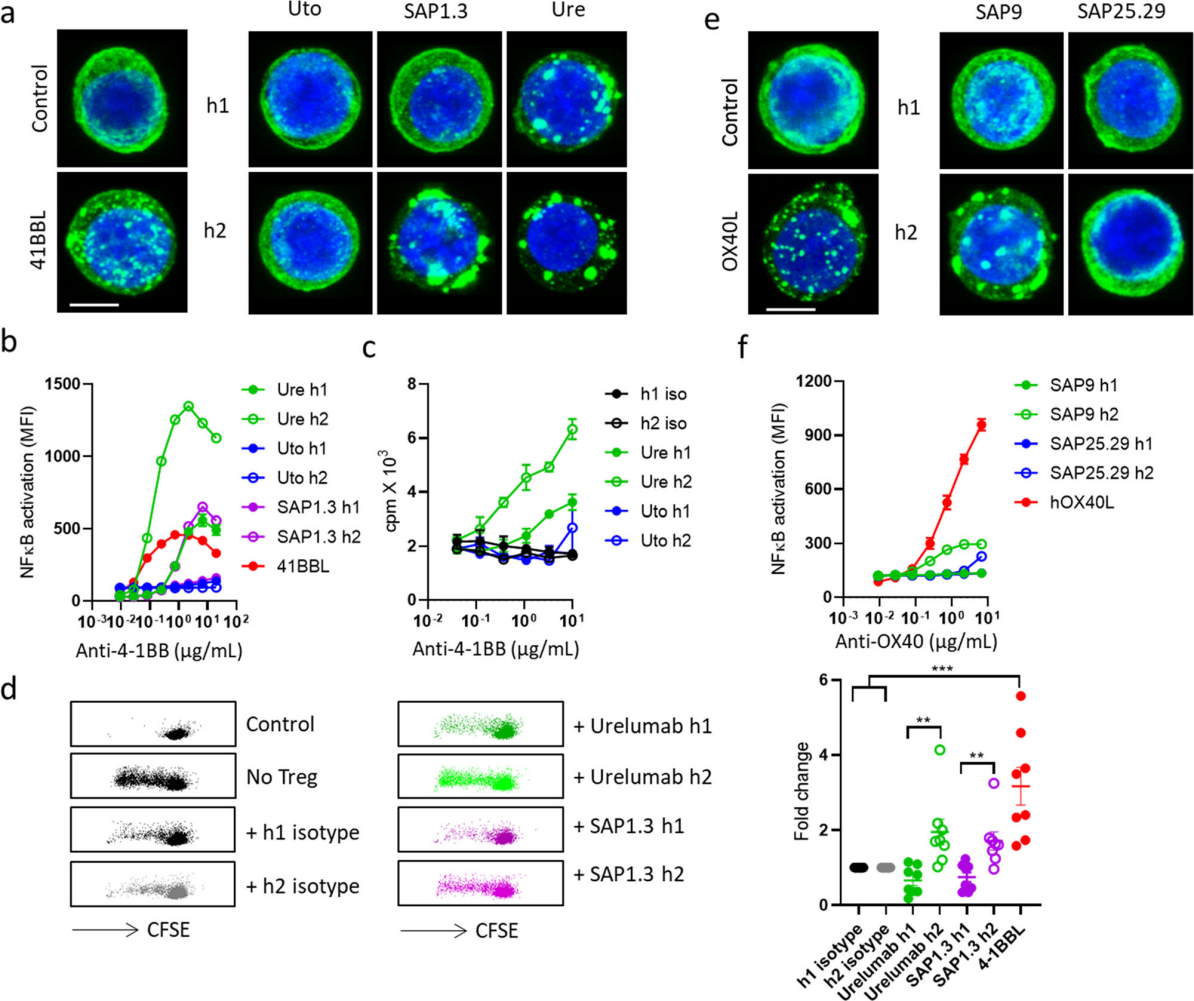
4-1BB and OX40 agonists induce differential receptor clustering. **a** Jurkat-h4-1BBECD-GFP cells were treated with 10 µg/mL anti-4-1BB mAbs of either h1 or h2 isotype as indicated for 1 h, and then fixed with methanol, nucleus stained with DAPI and imaged using a Leica SP8 confocal microscope. All images are representative of at least 10 images from at least 2 experiments. Scale bar, 4 µm. **b** Jurkat-NFκB-GFP reporter cells expressing h4-1BB were incubated with serially diluted anti-4-1BB mAbs as indicated for 6 h. The level of NFκB activation was then quantified by GFP expression assessed by flow cytometry. Means ± SEM, *n* = 3, data representative of 3 independent experiments. **c** Purified human CD8+ T cells were activated with plate-bound anti-CD3 mAb for one day and then treated with various anti-4-1BB mAbs for 1 or 2 days before ^3^H thymidine was added at 1 µCi per well for the last 18 h to assess T cell proliferation. **d** CD4+ CD127^low^CD25+ Tregs were purified from human PBMC and cocultured with CFSE-labelled human PBMC at a 1:4 Treg:PBMC ratio in the presence of suboptimal anti-CD3 mAb and various anti-4-1BB mAbs as indicated. Cells were incubated for 4 days and CD8+ T cell proliferation assessed by CFSE dilution using flow cytometry. Representative dot plots are shown. Right panel: mAb activity was normalised against isotype control and 4-1BBL activity was normalised against the average of both isotype controls. Each dot represents one donor. The paired Student *t* test for comparing h1 and h2 mAbs. One-way ANOVA followed by Tukey’s post hoc test for comparing 4-1BBL with isotype controls. Means ± SEM, *p < 0.05, **p < 0.01, ***p < 0.001. **e** Jurkat-hOX40ECD-GFP cells were treated with 10 µg/mL anti-OX40 mAbs of either h1 or h2 isotype as indicated for 1 h, and then fixed with methanol, nucleus stained with DAPI and imaged using a Leica SP8 confocal microscope. All images are representative of at least 10 images from at least 2 independent experiments. Scale bar, 4 µm. **f** Jurkat-NFκB-GFP reporter cells expressing hOX40 were incubated with serially diluted anti-OX40 mAbs as indicated for 6 h. The level of NFκB activation was then quantified by GFP expression assessed by flow cytometry. Means ± SEM, *n* = 3, data representative of 3 independent experiments.

To investigate whether the hIgG2-mediated differential receptor clustering is a conserved feature among TNFRs, we generated Jurkat cells stably expressing h4-1BB or hOX40, two additional important targets for cancer immunotherapy^3^. Similar to CD40, three different mAbs targeting h4-1BB induced differential receptor clustering, with hIgG2 conferring more prominent clustering than hIgG1 (Fig. 2a). Moreover, hIgG2 mAb stimulated higher levels of NFkB activation (Fig. 2b) and T cell proliferation (Fig. 2c) than hIgG1 and overcame Treg-mediated suppression of CD8 T cell proliferation (Fig. 2d). A similar correlation between clustering and agonism was shown for hOX40 mAb (Fig. 2e, f). Furthermore, FcγR over-expressing CHO-k1 cells were able to induce significant h4-1BB and hOX40 clustering and NFκB activation in an epitope-independent but isotype-dependent manner (Supplementary Fig. 5). Accordingly, clustering and agonism were afforded for hIgG1 mAb by CHO-k1 cells expressing any of the three FcγRs, whereas only FcγR2A-expressing CHO-k1 cells provided strong effects for hIgG2, in keeping with the known FcγR binding properties of these isotypes^34^.

Together, these data provide evidence from multiple agonists spanning three different TNFRs that functional agonism correlates with distinct patterns of receptor clustering, with hIgG2 resulting in more prominent clustering and agonism.

### Natural TNFR ligands and agonistic mAbs induce distinct receptor clusters

**Fig. 3:**
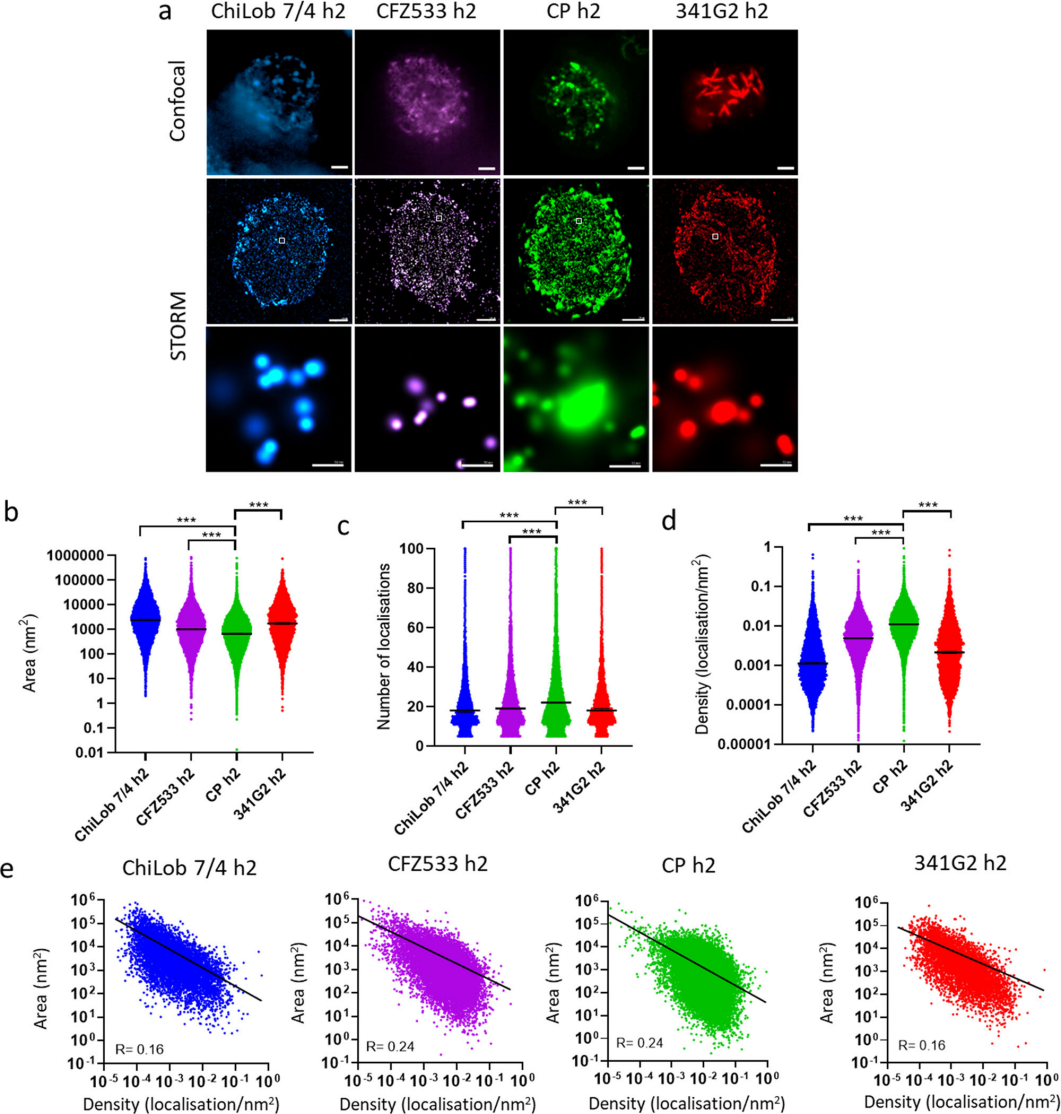
STORM assessment of differential CD40 clustering induced by a range of CD40 agonists. Jurkat cells expressing hCD40ECD-GFP were incubated with 50 µg/mL AF647-labelled anti-CD40 mAb at 37 °C for 1 h and then washed and fixed with 4% PFA. Cells were imaged using an ONI Nanoimager. **a** Representative confocal images (upper panel; scale bar, 2 µm), STORM images (middle panel; scale bar, 2 µm) and zoomed-in STORM image showing localisation patterns (lower panel; scale bar, 50 nm). Comparison of **b** area, **c** number of localisations and **d** density of clusters induced by various anti-CD40 mAbs as indicated, calculated by NimOS v1.6 software. Two-tailed, non-paired Student *t* test, *p < 0.05, **p < 0.01, ***p < 0.001. **e** Correlation between area and density of clusters induced by various anti-CD40 mAbs as indicated. Data are representative of three independent experiments.

The absence of receptor clustering correlated with a lack of agonistic activity (Figs. 1a and 2a–d), consistent with the paradigm that receptor oligomerization is required for TNFR activation. Our initial expectation was that the size of the receptor cluster and/or degree of receptor clustering would positively correlate with agonistic activity; with the strongest agonists displaying the largest and/or most extensive clusters. However, our confocal microscopy data indicated this was not correct. CD40 agonists displaying strong agonisms such as CP870,893 and CD40L induced notably smaller clusters than weaker agonists such as ChiLob 7/4 and SGN40 hIgG2 (Fig. 1a). Similarly, Urelumab hIgG2 evoked smaller clusters than SAP1.3 hIgG2 and yet was more agonistic (Fig. 2a, b, d). To investigate the receptor clustering features underpinning agonistic activity in more detail, we employed super-resolution stochastic optical reconstruction microscopy (STORM). Furthermore, to better preserve the native structure of receptor clusters, we fixed cells using a milder fixative, paraformaldehyde, instead of methanol. STORM-derived localisations were grouped into clusters using the hierarchical density-based spatial clustering of applications with noise algorithm (HDBSCAN), which revealed that receptor clusters observed under confocal microscopy further comprised substructures (Fig. 3a). The number of localisations detected within each cluster provided a quantitative measure of receptor abundance. We found that the strong agonist CP870,893 induced clusters characterised by small area and high receptor density compared with weaker agonists ChiLob 7/4 hIgG2 and CFZ533 hIgG2 (Fig. 3b, c, d). Interestingly, the cluster area exhibited an inverse correlation with receptor density for all mAbs (Fig. 3e). STORM analysis also identified a higher-order structure induced by the super-agonist 341G2 hIgG2 characterised by an elongated rod shape, which was absent in all other agonists characterised across different TNFRs (Fig. 3a).

### Receptor clustering causes epitope shielding

**Fig. 4:**
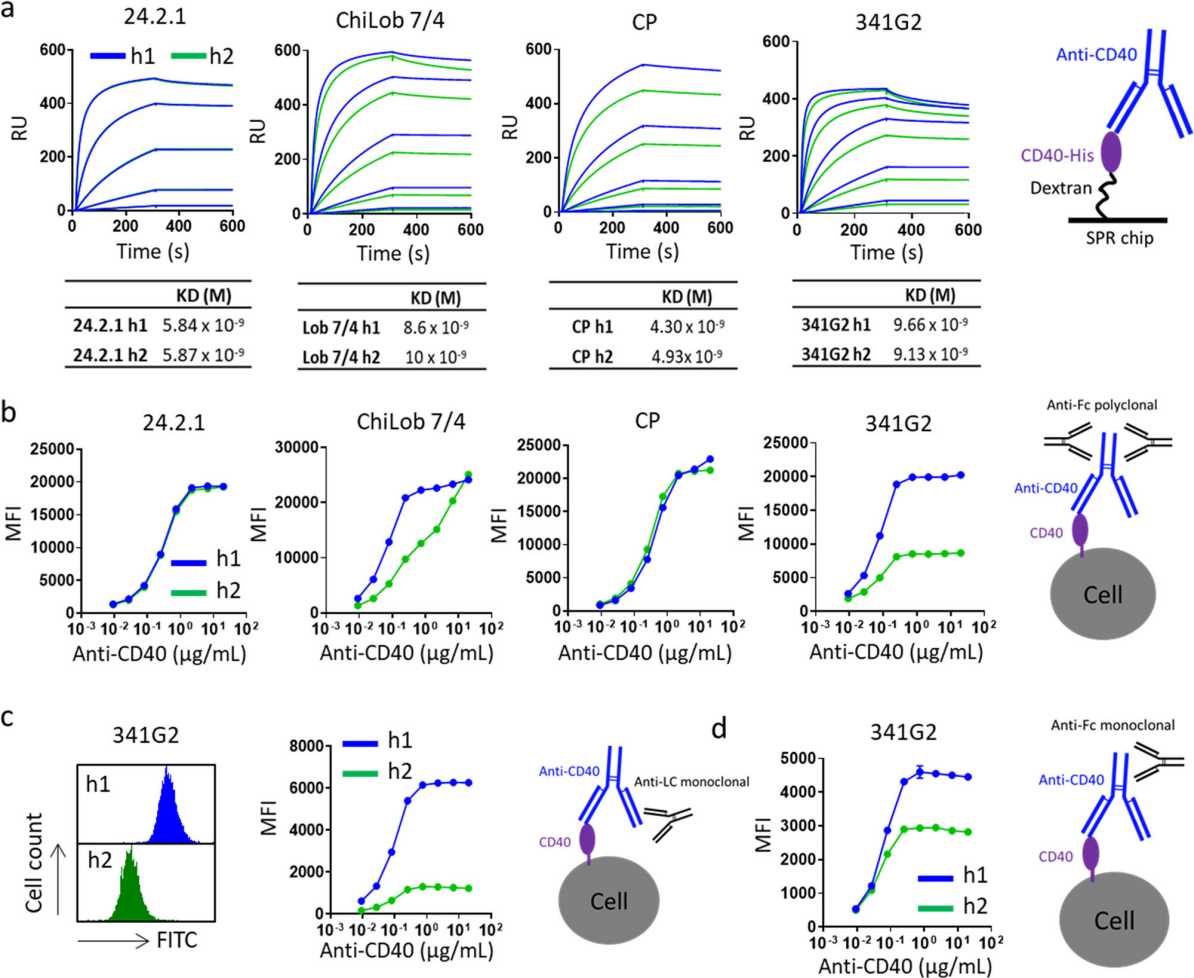
Differential receptor binding by anti-CD40 hIgG1 and hIgG2. **a** Recombinant hCD40ECD was immobilised onto CM5 chip via amine coupling and various anti-CD40 mAbs as h1 or h2 were injected at 250, 50, 10, 2, 0.4, and 0 nM using a Biacore T200 instrument. Both association phase and dissociation phase lasted 300 s. KD values were calculated using Biacore Bioevaluation software and values represent the mean from three independent experiments. **b** Ramos cells were incubated with various anti-CD40 mAbs as h1 or h2 as indicated and then washed and bound hIgG was detected by polyclonal anti-human IgG Fc, **c** monoclonal anti-human kappa light chain (left panel histograms represent cell surface binding of 341G2 h1 or h2 at 20 µg/mL) or **d** monoclonal anti-hIgG Fc. Means ± SEM, *n* = 3, data representative of independent 3 experiments.

To further understand the properties of receptor clusters driving powerful agonism, we took advantage of the unique properties of the hIgG1:hIgG2 anti-CD40 system; notably that these mAbs have defined binding epitopes, identical between hIgG1:hIgG2 pairs and yet distinct levels of clustering and agonism. Therefore, we selected a range of anti-CD40 mAbs across the agonism range (spanning hIgG2-conditional super-agonists such as 341G2 and isotype-agnostic-agonists such as CP870,893 as well as weak agonists such as ChiLob 7/4 and non-agonists such as 24.2.1) and compared their antigen-binding properties. Importantly, both hIgG1 and hIgG2 forms of each mAb bound similarly to CD40 when immobilised on an SPR chip (Fig. 4a). However, significant differences in binding to cell surface-expressed CD40 was observed for each mAb isotype pair as detected by secondary polyclonal anti-Fc antibody staining using flow cytometry (Fig. 4b). Notably, the hIgG2 variant of the super-agonist 341G2 bound significantly less than its hIgG1 variant whereas the non-agonist 24.2.1 showed equal binding for both isotypes (Fig. 4b). The reduced hIgG2 binding was also observed for ChiLob 7/4 but absent for CP870,893 (Fig. 4b). These differences largely recapitulated whether or not mAb induced large CD40 clusters (Fig. 1). Importantly, such differential binding was recapitulated when different secondary detection reagents (monoclonal anti-light chain (Fig. 4c) or anti-Fc (Fig. 4d)) were used, excluding epiphenomenon associated with a particular detection reagent. Similar to anti-CD40 mAb, the hIgG2 variants of mAbs targeting h4-1BB and hOX40 also exhibited reduced binding to cell surface-expressed h4-1BB and hOX40, respectively, according to their ability to induce receptor clustering (Supplementary Fig. 6a, b).

**Fig. 5:**
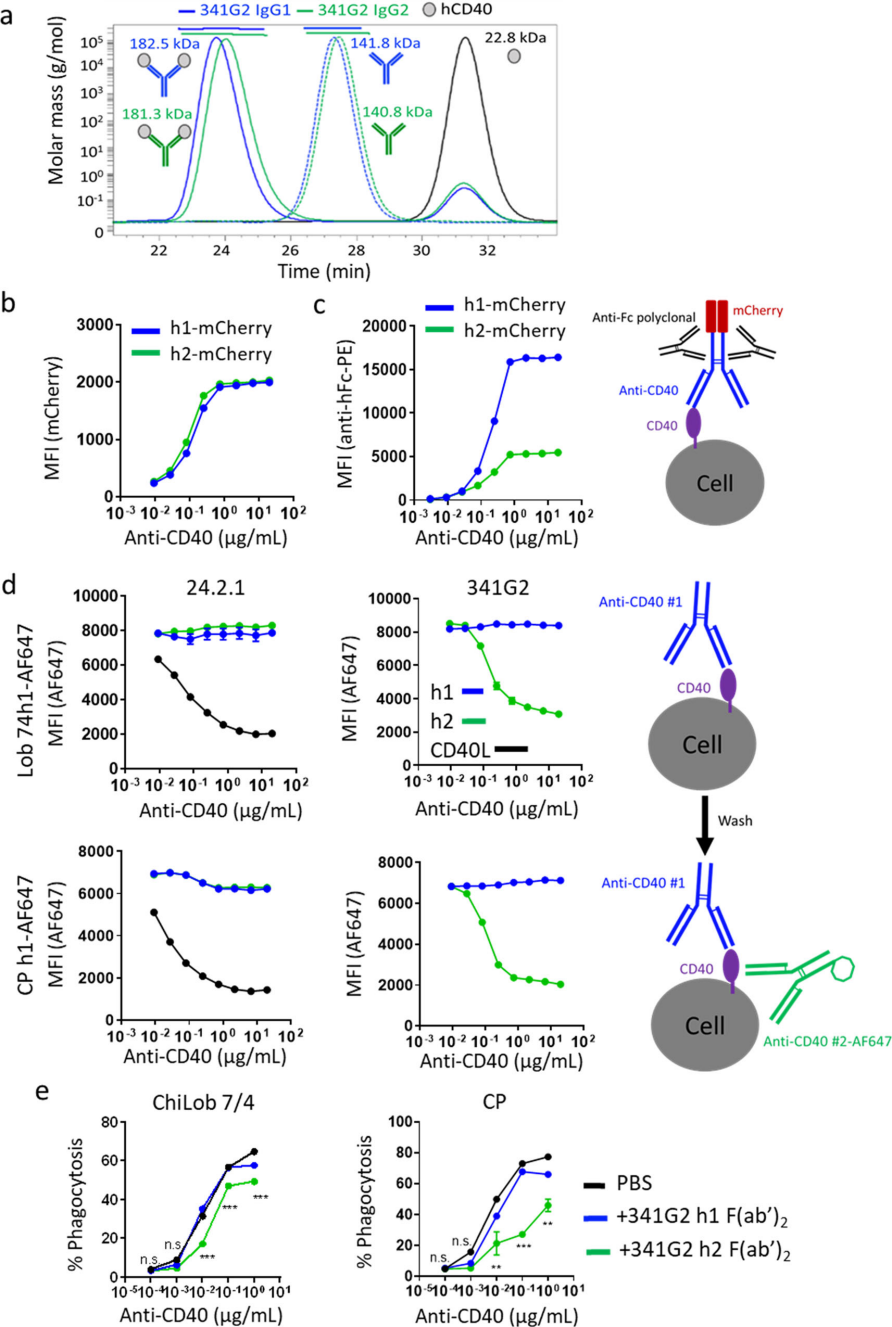
Receptor clustering results in epitope shielding. **a** Anti-CD40 mAb 341G2 h1 and h2 were co-incubated with recombinant soluble CD40ECD at room temperature for 30 min and then loaded onto a Superdex 200 HR10/30 column equilibrated with PBS and analysed by an in-line Dawn Heleos-II light scattering detector and an Optilab-rex refractive index monitor. Data analysis and molecular mass calculation were performed using ASTRA 6.1. The curve above each peak corresponds to the calculated molecular mass of each protein measured by the MALS. **b** Ramos cells were incubated with various concentrations of 341G2 h1-mCherry or 341G2 h2-mCherry and then washed and analysed for direct mCherry fluorescence by flow cytometry or **c** cells were further incubated with PE-conjugated polyclonal anti-hIgG Fc and then analysed for PE fluorescence by flow cytometry. **d** Ramos cells were pre-treated with unlabelled 341G2 or 24.2.1 h1, h2 or CD40L for 30 min before being washed and incubated with AF647-labelled ChiLob 7/4 h1 or AF647-labelled CP h1. AF647 fluorescence was detected by flow cytometry. Means ± SEM, *n* = 3, data representative of 3 independent experiments. **e** CFSE-labelled Ramos cells were pre-treated with 341G2 h1 F(ab’)_2_, 341G2 h2 F(ab’)_2_ or PBS control and then opsonized with various concentrations of ChiLob 7/4 h1 or CP h1 for 30 min before incubation with human monocyte-derived macrophages for 1 h for phagocytosis. CD14 was used to distinguish macrophages from Ramos cells. CD14+ CFSE+ cells represent macrophages that have undergone phagocytosis and were identified by flow cytometry. Means ± SEM, *n* = 2; two-way ANOVA followed by Sidak’s multiple comparisons were carried out for 341G2 h1 F(ab’)_2_ and 341G2 h2 F(ab’)_2_, *p < 0.05, **p < 0.01, ***p < 0.001, data representative of 2 independent experiments.

We hypothesised that such differences in receptor binding arose from isotype-specific differences in mAb-antigen binding stoichiometry; or that receptor clustering inherently shielded receptors from further molecular accessibility from secondary antibody detection. Size exclusion chromatography-multi-angle laser light scattering (SEC-MALS) allows the determination of the precise molecular weight of protein complexes, and showed that both hIgG1 and hIgG2 variants of 341G2 bound the same number of CD40 molecules in solution (Fig. 5a); discounting the first hypothesis. However, being in-solution, SEC-MALS is not entirely representative of cell surface binding due to the absence of steric factors. Therefore, to conclusively establish the level of mAb bound to the cell surface without the use of a secondary antibody, we generated anti-CD40 mAbs recombinantly fused with the fluorescent protein mCherry at the C-terminus of each heavy chain; thereby establishing the same 2:1 mCherry:mAb ratio for each mAb. Binding studies with these mAbs indicated that 341G2 hIgG1-mCherry and hIgG2-mCherry bound to the same extent on the cell surface (Fig. 5b); however, significantly less 341G2 hIgG2-mCherry was detected when using a secondary antibody, similar to results shown above with naked mAb (Fig. 5c). Together, these results indicate that receptor clustering impairs the ability of the secondary antibody to detect bound agonistic mAb.

We next hypothesised that agonist-induced TNFR clustering on the cell surface sterically hinders the receptor-agonist complex from further molecular access (e.g. from secondary detection agents). Indeed, in paraformaldehyde-fixed cells where receptor clustering is disabled due to prior receptor chemical cross-linking, the differential binding of hIgG2 versus hIgG1 341G2 no longer occurs (Supplementary Fig. 6c). This is also supported by the similar levels of hIgG1 and hIgG2 detected by Western blot where the non-covalent receptor–mAb complex was disrupted by detergent, permitting equivalent secondary antibody access (Supplementary Fig. 6d). To further test the hypothesis that receptor clustering causes epitope shielding, we pre-treated Ramos cells with the hIgG1 or hIgG2 variants of mAb designed to differentially induce receptor clustering (341G2) or not (24.2.1), and then added directly labelled-ChiLob 7/4 hIgG1 or CP870,893 hIgG1, which have different, non-competitive, binding epitopes than 341G2 and 24.2.1^8,31^ (Supplementary Fig. 7), to examine the effect of clustering on their binding. As shown in Fig. 5d, while the non-clustering hIgG1 variant of 341G2 did not influence the subsequent binding of ChiLob 7/4 or CP870,893, the cluster-inducing hIgG2 dose-dependently inhibited the binding of both ChiLob 7/4 and CP870,893, akin to the activity of CD40L (Fig. 5d), and resulted in reduced antibody-mediated phagocytosis (Fig. 5e). Moreover, the non-clustering 24.2.1 did not modulate ChiLob 7/4 and CP870,893 binding as either hIgG1 or hIgG2 (Fig. 5d). To explore further the clustering capacity of the hIgG2 mAb and its subsequent ramifications, we considered its natural function within an immune response.

### The hIgG2 isotype confers high avidity antigen binding

**Fig. 6:**
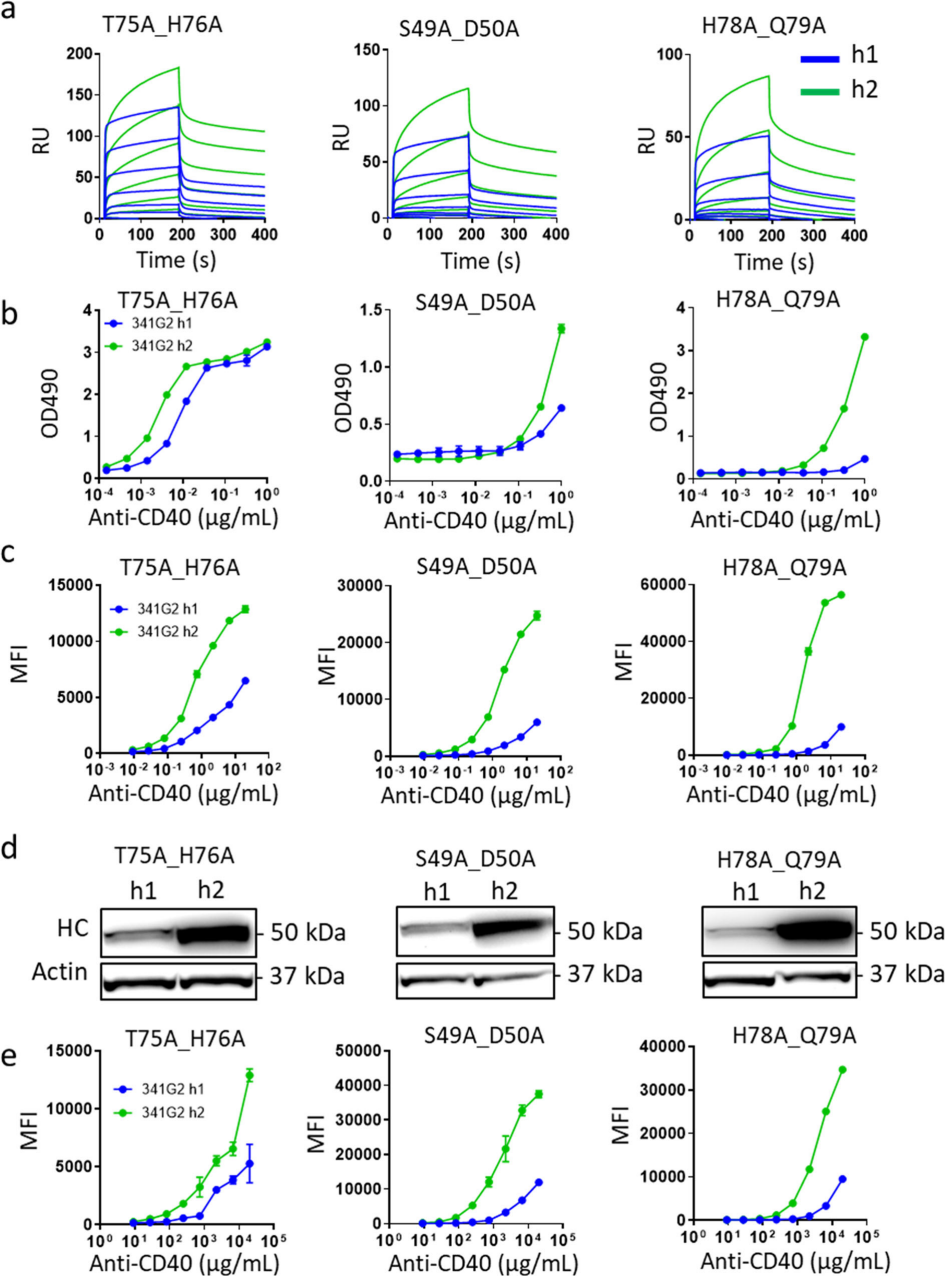
hIgG2 confers higher binding avidity to low-affinity antigen and exhibits self-association. **a** Recombinant soluble His-tagged CD40ECD mutants were captured by anti-His mAb pre-immobilised onto a CM5 chip, and then 341G2 h1 or h2 were injected at 1000, 333, 111, 37, 12.3, 4.1 or 0 nM using a Biacore T200 instrument. The association phase lasted for 180 s and the dissociation phase lasted for 300 seconds. **b** Recombinant soluble WT or mutant CD40ECD was coated onto ELISA plates at 5 µg/mL overnight; the next day, plates were washed and incubated with serially diluted 341G2 h1 or h2 for 1 h and then bound hIgG was detected by anti-hFc-HRP. Means ± SEM, *n* = 3. **c** CHO-k1 cells expressing various CD40 mutants on their surface as indicated were incubated with serially diluted 341G2 h1 or h2 for 30 min and then bound hIgG was detected by secondary anti-hFc-PE using flow cytometry. Means ± SEM, n = 3. **d** CHO-k1 cells expressing various CD40 mutants on their surface as indicated were incubated with 20 µg/mL 341G2 h1 or h2 for 30 min and then cells were lysed and the lysates subject to detection of hIgG by Western blotting as described in Methods. **e** CHO-k1 cells expressing various CD40 mutants on their surface as indicated were fixed with 4% PFA and then incubated with serially diluted 341G2 h1 or h2 for 30 min and bound hIgG was detected by secondary anti-hFc-PE using flow cytometry. Means ± SEM, *n* = 3. All data are representative of at least 3 independent experiments.

The hIgG2 isotype dominates the humoral response to bacterial capsular polysaccharides and is known for its low affinity, high avidity characteristics^35–37^. We, therefore, evaluated the potential impact of hIgG2 on anti-TNFR mAb avidity. As most anti-TNFR mAbs are selected for inherent high affinity for their targets, we utilised a series of CD40 mutants engineered to exhibit reduced affinity for the anti-CD40 mAb 341G2 to mimic the endogenous low-affinity hIgG2 response against polysaccharide. Surface plasmon resonance (SPR) shows that 341G2 hIgG1 and hIgG2 variants bound to the wild-type CD40 with similar kinetics; however, compared to hIgG1, hIgG2 bound significantly better to all three of the low-affinity CD40 mutants tested (Fig. 6a). SPR sensorgrams indicated that the hIgG2 mAb binding to the low-affinity mutants comprised two distinct phases: an initial fast association phase shared by hIgG1, followed by a slow secondary association phase largely absent with hIgG1. A similar two-step dissociation phase, characterised by an initial fast off-rate followed by a slower off-rate, was also apparent (Fig. 6a). Notably, hIgG2 binding did not reach a plateau at the end of the 200-second association phase (Fig. 6a); therefore, we extended the length of association by 10 fold and found that hIgG2-associated binding continued to increase with time, leading to more divergent binding from hIgG1 (Supplementary Fig. 7b), indicating self-association. To exclude the possibility of existing antibody aggregate contributing to this phenomenon, we repeated experiments in which even more stringent size exclusion chromatography was performed to elicit nearly 0% aggregate, including immediate post-purification SPR analysis, and obtained identical results. For these reasons, we are confident these observations reflect dynamic self-association of individual hIgG2 molecules. Furthermore, the addition of hIgG2 Fc fragments, incapable of binding CD40, did not significantly impact binding to the SPR chip, either alone or in the presence of 341G2 h2, indicating that the observed self-association requires antigen binding. In contrast, 341G2 hIgG1 reached a plateau early during the association phase (Supplementary Fig. 7b). Such enhanced, hIgG2-mediated binding for low-affinity CD40 mutants was recapitulated by enzyme-linked immunosorbent assay (ELISA) (Fig. 6b). Furthermore, we generated cell lines transfected with these CD40 mutants to test if this hIgG2-mediated effect was also evident on the cell surface. Consistent with SPR and ELISA data, hIgG2 bound these cells significantly better than hIgG1 as assessed by flow cytometry (Fig. 6c) and Western blot (Fig. 6d). Moreover, the augmented binding was maintained when cells were pre-fixed, indicating that the enhanced effect is independent of receptor rearrangement on the cell surface (Fig. 6e).

## Discussion

The human TNF–TNFR superfamily comprises 29 TNFRs and 19 TNF ligands that regulate many fundamental biological processes^14,15^. Genetic mutations within this superfamily lead to diseases such as cancer, autoimmunity and immune deficiency^38^. Conversely, therapeutic exploitation of the TNF–TNFR axis has led to effective drugs such as adalimumab and etanercept for autoimmune diseases as well as promising antitumor agents targeting the costimulatory immune receptors^1,2^. Current approaches in the latter category include stimulating antigen-presenting cells, reprogramming suppressive myeloid cells, activating cytotoxic T cells and selective depletion of regulatory T cells (Treg)^3,32,39^. Understanding the mechanism of TNFR activation and downstream signalling is key to devising optimal therapeutic interventions in each case.

Receptor oligomerization represents the paradigm for TNFR activation^4^ but the precise molecular process underpinning agonism is unclear. TNFRs lack intrinsic cytoplasmic enzymatic activity and rely on TRAFs or death domain-containing adaptors for downstream signalling^4^. While TNF ligands are predominantly trimeric, the oligomeric state of ligand-free TNFRs at the cell surface remains unclear. Apart from the prototypical TNFR1 shown to be dimeric^40^, crystal structures of other TNFRs such as 4-1BB, RANK, DcR3 and DR6 do not support a universal dimeric state^20,41–43^. Moreover, crystal structures of TNFR-ligand complexes reveal that most TNFRs form a trimeric complex with their cognate ligand^4^. On the cell surface, various TNFRs including TNFR1, TNFR2, Fas, DR4 and CD40 have been found as pre-assembled, non-covalent, dimers and trimers formed via homotypic interaction between the PLAD in CRD1^24–26^. Such PLAD is supported by experiments involving biochemical cross-linking of cell surface receptors, FRET, and more recently by super-resolution microscopy^29^. However, evidence for PLAD-PLAD interaction between recombinantly expressed CRD1 domains remains unclear, likely due to its low-affinity interaction rendering routine biophysical measurements difficult^30^. The idea that each PLAD serves as a receptor-specific regulatory element for self-assembly offers a mechanism for the differential sensitivity of TNFRs to various forms of agonist. For example, soluble TNF effectively activates TNFR1 but not TNFR2 despite efficient binding to both receptors^44,45^.

The clinical significance of PLAD was demonstrated in autoimmune lymphoproliferative syndrome (ALPS). In ALPS, patients are only heterozygous for certain deleterious Fas mutations, yet exhibit greatly impaired lymphocyte apoptosis, leading to autoimmunity. Subsequent studies showed that the various ALPS mutations map to all parts of Fas except CRD1 and an intact CRD1 of a mutant dysfunctional Fas molecule is required to allow association with wild-type Fas via the PLAD to disable its signalling^24^.

Establishing the oligomeric state of TNFRs is crucial for understanding the molecular mechanism of TNFR activation. The early sequential model predicted that a trimeric ligand initially engages one receptor monomer before sequentially recruiting additional receptors to form a trimer^4^. The two-step model supersedes this by providing PLAD-mediated higher-order oligomerization of the initial receptor-ligand complex in adjacent complexes^4^. A more recent model, based on the dimeric TNFR1 crystal structure, proposes that ligand-free TNFR dimers on the cell surface form hexagonal arrangements which expose three receptor binding sites to the trimeric ligand^14^. Applying super-resolution microscopy to additional TNFRs will help establish the broader significance of pre-ligand receptor assembly among the TNFR superfamily members^29,46^.

To date, there remains no crystal structure of a bivalent mAb-TNFR complex due to technical challenges associated with the size of the complex and the flexible IgG hinge. However, a wealth of information has been generated on the functional requirements of agonistic mAb. For example, we previously demonstrated that the complex interplay between epitope, isotype and FcγR availability determines the agonistic activity of anti-CD40 mAbs, and similar principles are being applied to other TNFRs^7–9,47^. Fluorescence microscopy has shown that TNF ligands and agonistic mAbs induced TNFR clustering on the cell surface; however, the requirement for secondary crosslinking remains unresolved^46,48–50^. For example, both secondary crosslinking-dependent and independent clustering of Fas were reported for soluble FasL and agonistic anti-Fas mAb^51–53^. Such discrepancies may result from the different qualities of recombinant proteins and cell types used. Irrespective of these technical issues, mechanistic insight into TNFR activation remains limited beyond a known blanket requirement for receptor clustering, with data generated to date indicating a binary relationship between clustering and agonistic activity, and a detailed mechanism to account for the differential agonism exhibited by multiple mAb agonists remains lacking.

In this study, we employed a large panel of clinically relevant CD40 agonists exhibiting different isotypes, known to mediate differential levels of receptor agonism to explore this question in the absence of complications arising from the use of secondary crosslinking through additional antibodies, soluble receptors or cell surface receptors such as FcγR. Confocal microscopy indicated that the hIgG2 variant of ChiLob 7/4, CFZ533, SGN40 and 341G2 induced greater receptor clustering compared with hIgG1, correlating with their enhanced agonistic activity. Similar hIgG2-facilitated receptor clustering and functional activity were evident for anti-4-1BB and anti-OX40 mAbs, suggesting a conserved mechanism of hIgG2-mediated enhancement among TNFRs. Non-agonistic mAbs failed to induce appreciable receptor clustering, whether as inert epitopes or inappropriate isotypes. These data confirm the absolute requirement for receptor clustering to drive agonism. The explicit isotype-conditional agonism demonstrated with 341G2 (highly agonistic as hIgG2 and totally inert as hIgG1) infers that Fab-induced conformational change is not responsible. Moreover, a unique higher-order structure was observed for the super-agonist 341G2 hIgG2. We previously observed both rounded and rod-shaped CD40 clusters induced by 341G2 hIgG2 when cells were fixed with methanol^31^. In the current study, rod-shaped clusters represented the dominant structure when the milder fixative paraformaldehyde was used. While the mechanism remains unclear, the 341G2 epitope, unusually spanning both CRD1 and CRD2, may promote CD40 clustering in an unconventional geometry that supports this molecular arrangement leading to super-agonism. It should also be noted that although in our experience the hIgG2 isotype has evoked agonism for CD40 and other TNFRs, there are examples where the heavy chain of an anti-TNFR2 mouse IgG2b has been replaced with that of hIgG2 to provide a more powerful antagonist^54^. It remains unclear if the Fc domain of these chimeric antibodies are required for their antagonistic function but indicates that the underpinning clustering geometry is important in each case.

Biologically, hIgG2 represents the dominant IgG isotype produced in response to carbohydrate antigens such as bacterial capsular polysaccharide and blood group antigens; however, the functional significance of this isotype bias remains unresolved^55–57^. Carbohydrate-specific antibodies generally possess lower affinity due to a lack of T cell help during affinity maturation, which is thought to be compensated for by the repetitive nature of bacterial capsular polysaccharide, providing a structural basis for increased avidity^36,37^. Therefore, we tested whether hIgG2 could similarly enhance avidity for TNFRs, by engineering mutated CD40 molecules with reduced affinity for 341G2 to mimic low-affinity anti-carbohydrate antibodies. Consistent with the notion that hIgG2 enhances antibody avidity for low-affinity antigens, 341G2 hIgG2 exhibited significantly higher binding to the CD40 mutants than hIgG1 in multiple assay formats. SPR sensorgrams indicated a biphasic association and dissociation for hIgG2. Moreover, hIgG2 binding failed to reach saturation despite an extended association phase, which suggests self-association. Having observed such apparent self-association, we performed more stringent size-exclusion chromatography to ensure that this was not due to pre-existing antibody aggregate but instead represents an intrinsic hIgG2-associated property.

The cross-species examination has identified mouse IgG3 and rat IgG2c as hIgG2 counterparts based upon their similar dominance in the anti-carbohydrate humoral response in vivo, and observations that mouse IgG3 was found to mediate similarly enhanced binding to carbohydrate antigens^58–61^. Human IgG self-association upon antigen binding has also previously been reported for hIgG1 and hIgG4^62–65^. Our finding that hIgG2 conferred high avidity for low-affinity TNFR variants offers an insight into the mechanism of hIgG2-mediated receptor clustering and agonistic activity and such self-association provides a mechanism through which additional receptor clustering could nucleate.

In addition to the strong hIgG2-mediated receptor clustering described above for 341G2, we observed that strong agonists CP870,893 and CD40L induced noticeably smaller clusters than the weaker hIgG2 agonists ChiLob 7/4, CFZ533 and SGN40, which challenges the general notion that larger receptor clusters elicit stronger agonism. Further examination by STORM microscopy revealed that clusters induced by CP870,893 exhibited smaller, higher density receptor clusters than the weaker agonists. Small clusters with higher receptor density presumably allow more efficient recruitment and activation of TRAFs for downstream signalling. Crystal structures of the TRAF domain of human TRAF1–5 demonstrate a conserved trimeric mushroom-shaped structure comprising the TRAF-C domain that mediates interaction with TNFRs and the elongated TRAF-N domain which serves as the binding site for various signalling adaptors^66–70^. The trimeric TRAF domain matches the trimeric state of most TNFR-ligand complexes, the basic unit of TNFR signalling, raising the possibility that the trimeric signalosome evolved for optimal TRAF-mediated signalling. Larger receptor clusters could potentially cause TRAF-N domain “overcrowding” that sterically hinders efficient recruitment of signalling adaptors, in a manner akin to receptor clustering-induced epitope shielding. Interestingly, our STORM analysis revealed a trend for smaller receptor clusters to possess higher receptor density, suggesting that small clusters are more amenable to receptor oligomerization and subsequent signalling. Given the complexity of TNFR signalling, it is likely that multiple factors including the cluster number, size and density combine to determine the magnitude of cellular response. Indeed, it is also clear that there are multiple ways to achieve effective clustering of the TNFRs to elicit downstream signalling (Supplementary Fig. 8).

We also found that agonism-mediating receptor clustering could lead to epitope shielding, due to the sequestration of the receptor-agonist complex from further external molecular access. Moreover, it is possible that the orientation of the Fab arm hinders the accessibility of the secondary antibody leading to reduced apparent detection^71^. Given the short 30-min incubation during relevant assays and our previous report that receptor clustering did not lead to significant CD40 internalisation over this period, it is unlikely that the observed epitope shielding on the cell types studied was due to receptor internalisation. Indeed, CD40 was reported to be less endocytic than other receptors such as mannose receptors, DEC205 and CD20^72,73^. Nevertheless, we could not completely exclude the possibility of antibody-mediated CD40 internalisation. Epitope shielding could represent a simple unevolved consequence of clustering-induced steric exclusion. However, as ligand-induced receptor clustering is a common mode of activation among other receptor classes such as receptor tyrosine kinases^74^, it is possible that such sequestration of receptor–agonist complex represents a regulatory mechanism for cellular activation. For example, some TNFRs have multiple cognate ligands^75^; upon specific ligand-induced receptor activation, epitope shielding could reduce receptor accessibility for other ligands to prevent unwanted signalling. While we did not find previous reports describing the phenomenon of clustering-induced epitope shielding, it could potentially explain some reported technical caveats related to flow cytometry. For example, when Foxp3–GFP transgenic Treg cells underwent vaccine-induced reprogramming, the Foxp3 protein became difficult to detect by flow cytometry using secondary antibodies but remained readily detectable by GFP fluorescence and Western blotting^76^. It is possible that Treg activation leads to Foxp3 clustering/sequestration rendering it less amenable to detection by secondary antibodies.

In summary, our work demonstrates that anti-TNFR agonists mediate a range of agonistic activities through distinct forms of receptor clustering. Strong agonists induced smaller clusters with higher receptor density, while the wild type hIgG2 isotype enhances the clustering of CD40, 4-1BB and OX40, through a mechanism that may involve improved antibody avidity. Furthermore, CD40 super-agonism was manifested alongside higher-order rod-shaped CD40 clusters. This work sheds light on the fundamental mechanism of TNFR activation and knowledge of the receptor sub-structures required for different levels of agonism, thereby guiding the development of future TNFR agonists.

## Methods

### Mice

hCD40 transgenic mice (hCD40Tg) were provided by Professor Randolph Noelle (King’s College, London)^77^. hCD40Tg/FcγR null mice (hCD40Tg*/Fcer1g*^*−/−*^/*FcγR2b*^*−/−*^) were generated as previously described^31^. Briefly, homozygous FcγR null mice (*Fcer1g*^*−/−*^ x *FcγR2b*^*−/−*^) were generated by breeding *Fcer1g*^*−/−*^ and *FcγR2b*^*−/−*^ mice^78^, and hCD40Tg/FcγR null mice were generated by crossing hCD40Tg mice with homozygous FcγR null mice. All animals were maintained and bred in-house. Mice were maintained on a 12 h light/dark cycle, food and water was made available at all times, environmental enrichment was provided and temperature was maintained between 20-24  °C. Mice were visually checked daily to ensure healthy status. All experiments were conducted under UK Home Office licence numbers PB24EEE31, P4D9C89EA, P540CBA98 and P39FE2AA7 and according to local ethical committee guidelines.

### Human samples

Human PBMCs were isolated from healthy donor leucocyte cones obtained through Southampton National Blood Services with prior informed consent, and the use of human blood was approved by the East of Scotland Research Ethics Service, Tayside, UK.

### Cell lines

Wild type Jurkat, Ramos and CHO-k1 cell lines were obtained from ATCC and maintained in a humidified incubator at 37 °C and 5% CO_2_, cultured in RPMI supplemented with 10% heat-inactivated FBS (Sigma), 2 mM l-glutamine, 1 mM pyruvate, 100 U/mL penicillin, 100 µg/mL streptomycin (all from Thermofisher); Jurkat and Ramos cell culture media were additionally supplemented with 50 µM β-mercaptoethanol (Sigma).

### Antibodies and reagents

All anti-CD40 mAbs were generated as previously described^8,31^. ChiLob 7/4, Lob 7/6, SAP1.3, SAP9 and SAP25.29 were generated in-house by conventional hybridoma method and their variable domain sequences were amplified using PCR to enable isotype switching. The variable domain sequences of 24.2.1 (US2009/0130715A1), 341G2 (US8716451B2), CP870,893 (US20090130715A1), ADC1013 (WO2016023960A1), APX005M (WO2014070934A1), SGN40 (WO2007075326A2), CFZ533 (WO2012075111A1), Urelumab (WO2010/042433A1), Utomilumab (WO2015/119923A1) and TGN1412 (US7585960B2) were derived from published patents. The light and heavy chain variable domain sequences were then synthesised by GeneArt and cloned into the pEE12.4 and pEE6.4 expression vectors (Lonza, UK) engineered to encode different IgG isotypes. To generate 341G2 hIgG1-mCherry (h1) and 341G2 hIgG2-mCherry (h2), full-length mCherry (Accession number MF169983) was fused to the C-terminus of each heavy chain without any linker. Antibodies were produced in ExpiCHO cells by transient expression, purified by MabSelect Sure column (GE Healthcare, UK) and checked to contain < 1% aggregate by HPLC and < 5EU endotoxin/mg antibody using the Endosafe-PTS portable test (Charles River Laboratories, L’Arbresle, France). The amino acid sequence of the wild type hIgG2 CH1-CH3 domains used is

ASTKGPSVFPLAPCSRSTSESTAALGCLVKDYFPEPVTVSWNSGALTSGVHTFPAVLQSSGLYSLSSVVTVPSSNFGTQTYTCNVDHKPSNTKVDKTVERKCCVECPPCPAPPVAGPSVFLFPPKPKDTLMISRTPEVTCVVVDVSHEDPEVQFNWYVDGVEVHNAKTKPREEQFNSTFRVVSVLTVVHQDWLNGKEYKCKVSNKGLPAPIEKTISKTKGQPREPQVYTLPPSREEMTKNQVSLTCLVKGFYPSDIAVEWESNGQPENNYKTTPPMLDSDGSFFLYSKLTVDKSRWQQGNVFSCSVMHEALHNHYTQKSLSLSPGK

Recombinant soluble wild type and mutant CD40ECD domains were generated as previously described^31^. Briefly, site-directed mutagenesis was performed using QuickChange Site-Directed Mutagenesis Kit (Agilent, UK). DNA encoding the His_6_-tagged CD40ECD domain was cloned into pcDNA3.1 vector and transfected into ExpiCHO cells for 7 days before purification using Ni Excel column (GE Healthcare) followed by gel filtration using HiLoad 26/600 Superdex 200 pg (GE Healthcare). Recombinant trimeric CD40L, OX40L and 4-1BBL were produced in house. DNA encoding human CD40L (Met113-Leu261), human OX40L (Gln51-Leu183) or human 4-1BBL (Arg71-Glu254) fused with a FLAG tag and GCN4 leucine zipper motif via a (G3S)3 linker at the N-terminus were synthesised by GeneArt and subcloned into the pDSG104 vector (IBA Life Sciences, Germany). The plasmids were transfected into MEXi-293E cells (IBA Life Sciences, Germany) for 7 days before purification by anti-FLAG Affinity Gel (Sigma, UK) or Strep-Tactin (IBA Life Sciences, Germany). Antibodies and recombinant proteins were labelled with AF488 or AF647 using the Alexa Fluor™ 488 Antibody Labelling Kit or Alexa Fluor™ 647 Antibody Labelling Kit, respectively (Thermo Fisher Scientific, UK).

DNA encoding the IgG-degrading enzyme of Streptococcus pyogenes (IdeS) (Asp30-Asn341) was synthesised by GeneArt, cloned into the pOPINJ vector (Addgene) and transformed into JM109 bacteria (Promega) for plasmid amplification. For IdeS expression the pOPINJ vector was transformed into the Rosetta 2 (DE3) placI bacteria (Merck) and colonies were picked and cultured in Terrific Broth media supplemented with 1% glucose, ampicillin and chloramphenicol (Melford) at 37 °C. When the optical density of bacterial culture reached 1.0, 0.5 mM isopropyl β-d-1-thiogalactopyranoside was added for 4 h to induce protein expression. Bacteria were then harvested and the pellet lysed with primary amine-free Bugbuster supplemented with DNAse I, MgCl_2_ and lysozyme (all from Merck). IdeS was purified from the supernatant using the Glutathione 4B sepharose beads (GE Healthcare) according to the manufacturer’s recommendations and the purity was checked using SDS-PAGE. IgG F(ab’)_2_ was generated by IdeS digestion of the whole IgG for 1 h at 37 °C and then purified by gel filtration. The size and purity of F(ab’)_2_ was verified by SDS-PAGE. Anti-CD40 IgG Fab fragments were generated by digestion with Immobilised Papain (Thermo Fisher Scientific) according to the manufacturer’s protocol and then purified by gel filtration. Recombinant hIgG2 Fc fragments were purchased from Sinobiological, China.

### Confocal microscopy

DNA encoding hCD40ECD-GFP, full-length hCD40-GFP, hOX40ECD-GFP or h4-1BBECD-GFP were cloned into pCIpuro vector and then transfected into Jurkat cells using the Nucleofector Kit V (Lonza) or into CHO-k1 cells using GenePorter (Amsbio, UK). Stable Jurkat clones were selected using 1 µg/mL puromycin and stable CHO-k1 clones were selected using 10 µg/mL puromycin. Normal human B cells and hCD40Tg splenic B cells were isolated from human PBMC and hCD40Tg splenocytes, respectively using magnetic negative selection kits (StemCell Technologies, UK). To generate human immature DCs, CD14+ monocytes were isolated from human PBMC by magnetic negative selection kit (Miltenyi Biotech, UK) and then cultured in media supplemented with 500 IU/mL IL-4 and 1000 IU/mL GM-CSF (both produced in-house) for 6 days as described before^79^. The identity of the DC was confirmed by surface expression of CD11c (anti-human CD11c, 15508856, ebioscience, 1/20) and DC-SIGN (anti-human DC-SIGN, 330106, Biolegend, used at 1/50) by flow cytometry (Supplementary Fig. 7c). Confocal microscopy was performed as previously described^31^. To assess the effect of mAbs on receptor clustering, cells were incubated with 10 µg/mL mAb for one hour at 37 °C and then fixed with methanol before DAPI staining of the nucleus. Confocal images were acquired using a Leica SP8 confocal microscope and analysed using Leica Application Suite X (both from Leica).

### Live cell wide-field fluorescence microscopy

CHO-k1 cells expressing hFcγR1A, hFcγR2A or hFcγR2B were generated as previously described^33,80^. Briefly, hFcγR1A and its associated gamma chain were encoded in pcDNA3 and pCIpuro vectors, respectively and stable clones were selected using 10 µg/mL puromycin and 1 mg/mL geneticin; hFcγR2A was encoded in pCIpuro and stable clones were selected using 10 µg/mL puromycin; hFcγR2B was encoded in pcDNA3 and stable clones were selected using 1 mg/mL geneticin. CHO-k1 cells expressing various hFcγRs were plated onto IBIDI glass-bottom chambers overnight and then labelled with 5 µg/mL Hoechst 33342 and 1 in 2000 diluted Cellmask Orange (both from Thermofisher) the next day. Jurkat cells expressing hCD40ECD-GFP, hOX40ECD-GFP or h4-1BBECD-GFP were opsonized with 10 µg/mL target-specific mAb, labelled with Hoechst 33342 and then incubated with CHO-k1 cells for 15 min before wide-field fluorescence imaging using an ONI Nanoimager (Oxford, UK). To assess membrane-bound CD40L-mediated CD40 clustering, human CD4 T cells were isolated from healthy donor PBMC by magnetic negative selection kit (StemCell Technologies, UK) and then activated with 0.081 nM PMA and 1.34 nM ionomycin (both from Biolegend, UK) for 6 h. Purified normal human B cells and human monocyte-derived DCs were labelled with 10 µg/mL ChiLob 7/4 h1-AF488, known not to cross-block the CD40L-CD40 interaction^8^, in order to visualise CD40. Activated CD4+ T cells were labelled with Cellmask Orange and then cocultured with normal human B cells, DCs or Jurkat-hCD40ECD-GFP cells for 20 min before wide-field fluorescence imaging using an ONI Nanoimager (Oxford, UK); all cell populations were labelled with Hoechst 33342. Three wide-field images were taken per field of view corresponding to filter channels detecting Hoechst 33342, Cellmask Orange and GFP, respectively as recommended by the manufacturer and were overlaid using Photoshop CS6 (Adobe).

### STORM microscopy

IBIDI glass-bottom chambers were washed successively with 70% ethanol, acetone and distilled water and then coated with poly-L-lysine (Sigma). Jurkat cells expressing hCD40ECD-GFP were incubated with 50 µg/mL AF647-labelled anti-CD40 mAb at 37 °C for 1 h and then washed and fixed with 4% PFA. Cells were imaged in IBIDI glass-bottom chambers pre-coated with poly-l-lysine. Immediately before STORM, the ONI BiCubed STORM buffer (ONI, UK) was added to the well. STORM images were acquired using the ONI Nanoimager equipped with a 640 nm laser and STORM1.6 software (ONI, UK). The illumination angle was set to 52° and exposure was set to 30 ms; 10,000 images were collected per field of view. Localisations were identified using the NimOS v1.6 software. Localisations were grouped into clusters using HDBSCAN^81^. The following features were extracted for each individual cluster: a number of localisations; density (localisations/area) and area (computed from the convex hull of the cluster).

### Surface plasmon resonance

To compare the binding kinetics of h1 and h2 variants of anti-CD40 mAbs 24.2.1, ChiLob 7/4, CP870,893 and 341G2 for WT hCD40, recombinant soluble hCD40ECD was immobilised onto a CM5 chip via amine coupling according to manufacturer’s protocol and mAbs were injected through the flow cells at 250, 50, 10, 2, 0.4 and 0 nM in HBS-EP+ running buffer at a flow rate of 30 µL/min; 300 s for association and 300 s for dissociation. For affinity calculations, sensorgrams were fitted with the bivalent analyte binding model and the Equilibrium dissociation constant KD was calculated using Biacore Bioevaluation software. To compare 341G2 h1 and h2 binding to low-affinity hCD40ECD mutants, anti-His mAb was first immobilised onto CM5 chips via amine coupling according to the manufacturer’s protocol and 0.5 µg/mL of His-tagged soluble hCD40ECD mutants were captured for 30 s. Afterwards, anti-CD40 mAbs were injected at 1000, 333, 111, 37, 12.4 and 4.1 nM; the association phase lasted for either 180 or 2100 s as specified in figure legends and the dissociation phase lasted for 300 s. The Biacore T200 instrument was used throughout, and all reagents and Biacore Bioevaluation software were from GE Healthcare, UK.

### Enzyme-linked immunosorbent assay

Recombinant soluble WT or mutant CD40ECD proteins were coated onto MaxiSorp ELISA plates (Thermofisher) overnight at 5 µg/mL in PBS; the next day, plates were blocked with 1% bovine serum albumin and serially diluted 341G2 h1 and h2 were added to each well and incubated for 1 h. Plates were then washed and bound hIgG was detected by goat anti-hIgG Fc-HRP (Abcam, UK). Plates were developed with *o*-phenylenediamine dihydrochloride substrate and absorbance at OD490 was measured using an Epoch microplate spectrophotometer (Biotek).

### Flow cytometry

Flow cytometry experiments were performed using FACSCalibur, FACSCanto II, or LSR Fortessa machines (all from BD Biosciences). Flow cytometry data analysis was performed using FCS Express software Version 3 (De Novo Software) or Flowjo (BD). FACS gating strategies are included in Supplementary Fig. 10.

### Assessment of antibody cell surface receptor binding

To compare anti-CD40 mAb h1 and h2 binding to CD40 expressed on the cell surface, Ramos or CHO-k1 cells expressing CD40 were incubated with serially diluted anti-CD40 mAb for 30 min at 37 °C and then washed and remaining bound hIgG detected by secondary PE-conjugated polyclonal goat F(ab’)_2_ anti-hFc (Abcam, UK, 1/100), AF647-conjugated monoclonal anti-human kappa light chain (Biolegend, 1/100) or FITC-conjugated monoclonal anti-hFc (clone SB2H2, in-house, 10 µg/mL). To study epitope shielding, Ramos cells were pre-treated with the h1 or h2 variant of 341G2 or 24.2.1 for 30 min and then washed and stained with 1 µg/mL AF647-labelled ChiLob 7/4 h1 or AF647-labelled CP h1. To compare anti-OX40 and anti-4-1BB mAb h1 and h2 binding to cell surface-expressed OX40 and 4-1BB, respectively, stable Jurkat clones expressing OX40ECD or 4-1BBECD were incubated with various mAbs for 1 h at 37 °C and then washed and bound hIgG was detected by secondary DL650-conjugated polyclonal goat F(ab’)_2_ anti-hFc (Abcam, UK, 1/100). Cell samples were analysed using flow cytometry.

### Size exclusion chromatography-multi angle light scattering (SEC-MALS)

Anti-CD40 mAb 341G2 and recombinant soluble CD40ECD were co-incubated at 1:2 ratio at room temperature for 30 min and then loaded onto a Superdex 200 HR10/30 column (GE Healthcare) equilibrated with PBS and analysed by an in-line Dawn Heleos-II light scattering detector (Wyatt Technologies, UK) and an Optilab-rex refractive index monitor (Wyatt Technologies). Data analysis and molecular mass calculation were performed using ASTRA 6.1 (Wyatt Technologies). The curve above each peak corresponds to the calculated distribution of molecular mass of each protein measured by the MALS.

### Western blotting

Totally, 10 × 10^6^ Ramos cells or 5 × 10^6^ CHO cells expressing various CD40 mutants were incubated with 20 µg/mL anti-CD40 mAbs for 30 min before being washed three times with PBS and lysed in RIPA buffer (150 mM NaCl, 1% Triton X-100, 0.5% Deoxycholate, 0.1% SDS, 50 mM Tris, pH 8) supplemented with 2 mM Na_3_VO_4_, 50 mM NaF, and 1× Protease Inhibitor Cocktail (Sigma, UK). Cell lysates were centrifuged and determined for their protein concentration using Bradford assay (Biorad, UK), and then 10 µg cell lysate was reduced and loaded onto 12% Bolt gel for SDS-PAGE (Thermofisher, UK). Proteins were then transferred to an iBlot 2 nitrocellulose membrane using the iBlot 2 Gel Transfer Device (both from Thermofisher) and the membrane was blocked using PBST 5% milk for 30 min. The membrane was then probed overnight at 4 °C with rabbit monoclonal anti-human IgG (clone EPR4421, Abcam, UK, 1/1000) and rabbit anti-β-Actin (clone 13E5, Cell Signalling Technology, UK, 1/1000) before detection using secondary goat anti-rabbit IgG-HRP (Abcam, UK, 1/5000). The membranes were developed using Immobilon Classico Western HRP substrate or Immobilon Western Chemiluminescent HRP Substrate (both from Sigma) and chemo-luminescence captured using the UVP Biospectrum Imaging System.

### NFκB assay

pCIpuro plasmids encoding the full-length hCD40, hOX40 (expressing the hCD40 intracellular signalling domain) or h4-1BB (expressing the hCD40 intracellular signalling domain) were transfected into the Jurkat-NFκB-GFP reporter cell line (System Biosciences, USA) by nucleofection (Lonza) and stable clones were selected using 1 µg/mL puromycin. To assess NFκB activation, Jurkat cells were incubated with various anti-CD40, anti-OX40 or anti-4-1BB mAbs in the presence or absence of CHO-k1 cells expressing hFcγR1A, hFcγR2A or hFcγR2B for 6 h at 37 °C and the degree of NFκB activation was quantified by GFP fluorescence using flow cytometry.

### Mouse B cell activation assay

B cells were purified from hCD40Tg/FcγR null mouse spleens by magnetic negative selection kit (StemCell Technologies, UK). Totally, 1 × 10^5^ B cells were incubated with various anti-CD40 mAbs at 10 µg/mL in 96-well round-bottom plates for 3 days and then analysed for surface expression of CD23 using PE-conjugated anti-mouse CD23 (clone B3B4, Biolegend) by flow cytometry.

### Human T cell proliferation assay

To assess the costimulatory activity of anti-4-1BB mAbs, CD8 T cells were isolated from human PBMC by magnetic negative selection (StemCell Technologies, UK) and activated with plate-bound anti-CD3 mAb (OKT3, in-house, plated at 5 µg/mL) for 1 day. Cells were then treated with various anti-4-1BB mAbs for one or two days before ^3^H thymidine (Perkin Elmer) was added at 1 µCi per well for the last 18 h to assess T cell proliferation. To study the ability of anti-4-1BB mAbs to overcome Treg-mediated suppression of T cell proliferation, Tregs were isolated from human PBMC using the Human CD4+ CD127^low^CD25+ Regulatory T Cell Isolation Kit (StemCell Technologies, UK) and then added to CFSE-labelled human PBMC at a 1:4 Treg:PBMC ratio in the presence of 0.4 ng/mL or 0.8 ng/mL anti-CD3 mAb (OKT3, in-house). Cells were incubated for 4 days and then assessed for CD8+ T cell proliferation by CFSE dilution using flow cytometry.

### Antibody-dependent cellular phagocytosis

Antibody-dependent cellular phagocytosis was performed as previously described^82^, using human monocyte-derived macrophages (hMDM) as the phagocyte and Ramos cells as the target. Briefly, hMDM were generated by culturing monocytes in the presence of 100 ng/ml M-CSF (in-house) for 6 days and then 1 × 10^5^ hMDM were plated onto 96-well flat-bottom plate overnight. The next day, Ramos cells were labelled with CFSE and then treated with 5 µg/ml 341G2 h1-F(ab’)_2_ or 341G2 h2-F(ab’)_2_ for 15 min at 37 °C before opsonization by various concentrations of ChiLob 7/4 h1 or CP870,893 h1 for 30 min at 4 °C. Totally, 5 × 10^5^ target Ramos cells were added to each well-containing hMDM and incubated for 1 h at 37 °C for phagocytosis. Samples were subsequently stained with anti-CD14-APC (Biolegend, 1/100) to distinguish hMDM from Ramos cells and assessed by flow cytometry. % Phagocytosis was calculated as: (CFSE+ CD14+ cells)/(total CD14+ cells) × 100.

### Statistics and reproducibility

Flow cytometry data analysis was performed using FCS Express software Version 3 (De Novo Software) or Flowjo (BD). All other data analysis was performed using GraphPad Prism 7.05 (GraphPad Software). A two-tailed, non-paired Student *t* test was used for most pairwise comparisons. One-way ANOVA followed by Tukey’s post hoc test or two-way ANOVA followed by Sidak’s test was used for multiple comparisons as specified in figure legends. Throughout ^*^p < 0.05, ^**^p < 0.01, ^***^p < 0.001, n.s. not significant. Reproducibility including technical replicates and independent biological experiments are stated in each figure legend.

### Reporting summary

Further information on research design is available in the Nature Research Reporting Summary linked to this article.

## Supplementary information

Supplementary Information

Reporting Summary

## Data Availability

All data supporting the conclusions of this manuscript are included in this manuscript and supplementary information. Full Western blotting images are included in Supplementary Fig. 9. FACS gating strategies are included in Supplementary Fig. 10.
