# Supplementary material for: TNF receptor agonists induce distinct receptor clusters to mediate differential agonistic activity
Source: Commun Biol. 2021 Jun 23;4:772. doi: 10.1038/s42003-021-02309-5 (PMC8222242; doi:10.1038/s42003-021-02309-5)
Supplement: Supplementary file 1 — Supplementary Information [file 42003_2021_2309_MOESM1_ESM.pdf]

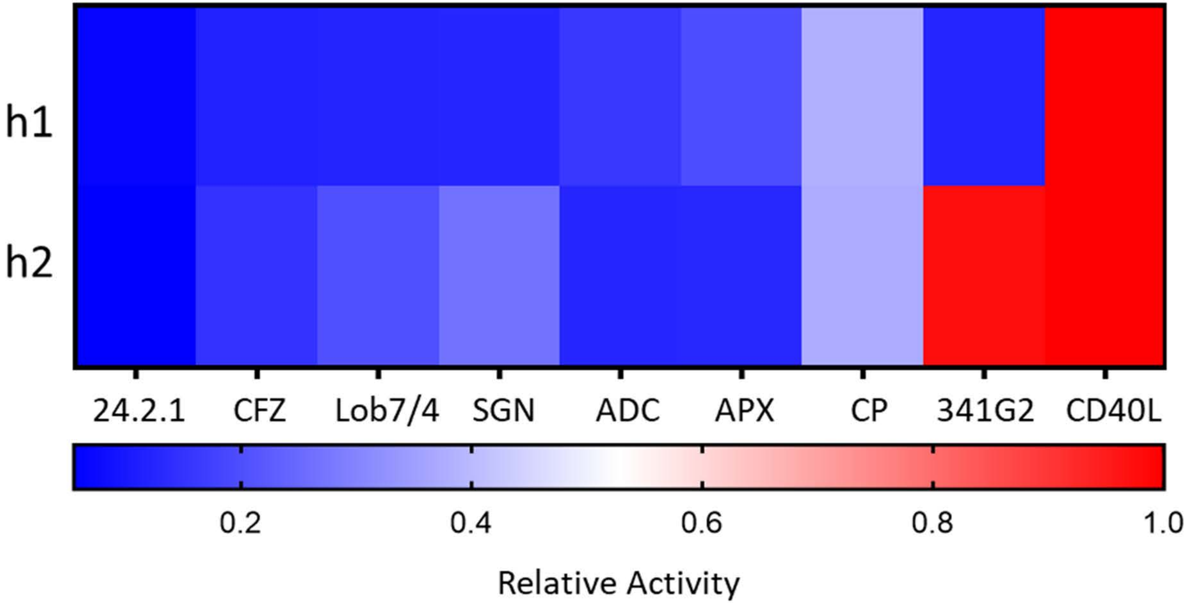
h1
h2
24.2.1
CFZ
Lob7/4
SGN
ADC
APX
CP
341G2
CD40L
0.2
0.4
0.6
0.8
1.0
Relative Activity

Supplementary Figure 1. Heat map ranking of the agonistic activity of CD40 agonists based on published human B cell proliferation data[1]. From left (blue) to right (red): low to high agonistic activity.

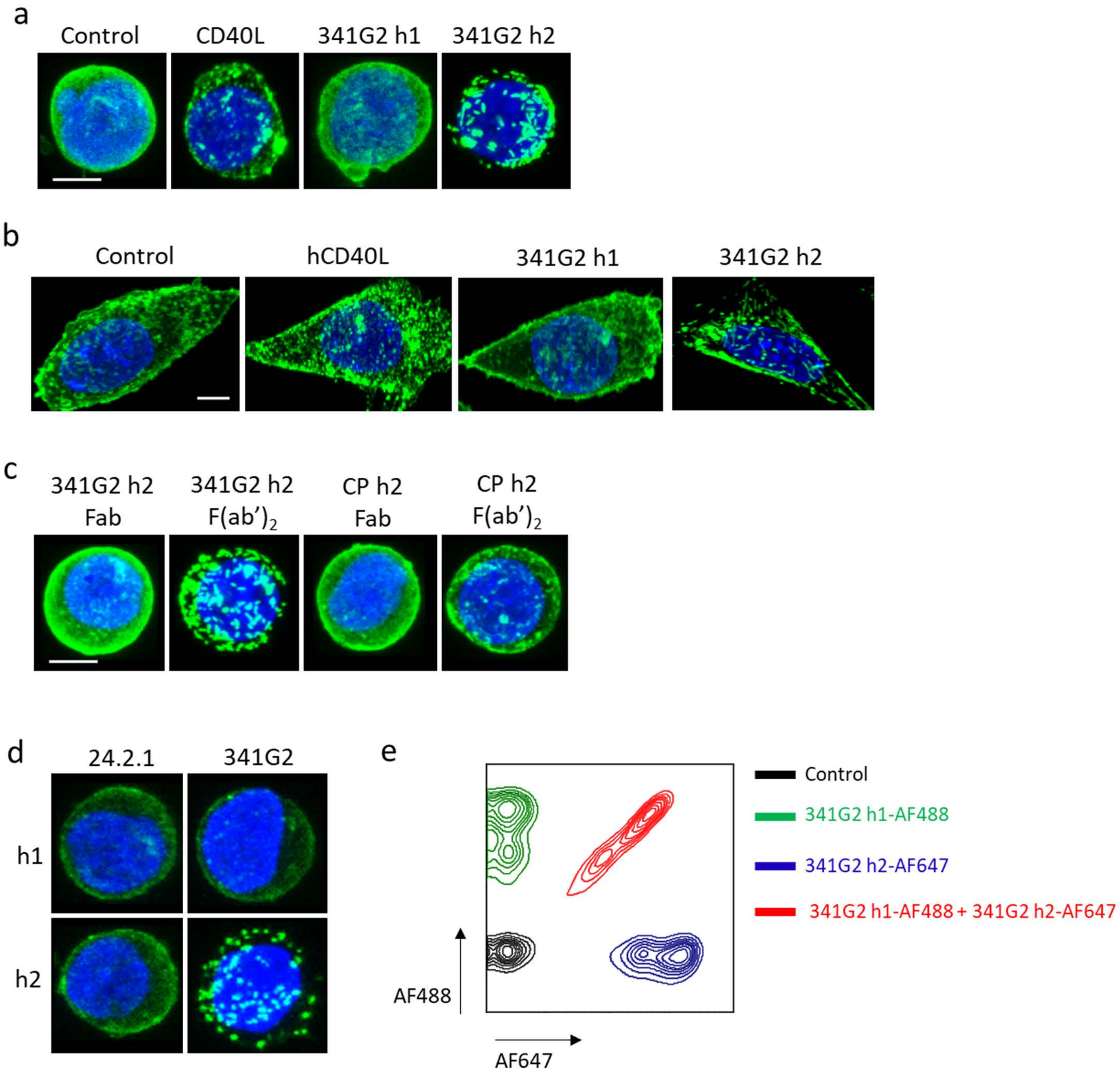

a
Control
CD40L
341G2 h1
341G2 h2
b
Control
hCD40L
341G2 h1
341G2 h2
c
341G2 h2 Fab
341G2 h2 F(ab')2
CP h2 Fab
CP h2 F(ab')2
d
24.2.1
341G2
h1
h2
e
AF488
AF647
Control
341G2 h1-AF488
341G2 h2-AF647
341G2 h1-AF488 + 341G2 h2-AF647

Supplementary Figure 2. CD40 agonists induce receptor clustering in different cell lines. a Jurkat cells expressing full length hCD40-GFP, **b** CHO-k1 cells expressing hCD40ECD-GFP, **c** Jurkat cells expressing hCD40ECD-GFP, and **d** Ramos cells were treated with 10 µg/mL of different CD40 agonists as indicated for 1 hour and then fixed with methanol, the nucleus stained with DAPI and imaged using a Leica SP8 confocal microscope. All images are representative of at least 10 images from at least 2 independent experiments. Scale bar, 4 µm. **e** Jurkat cells expressing hCD40ECD were incubated with 10 µg/mL 341G2 hIgG1-AF488, 341G2 hIgG2-AF647 or a mixture of both, and bound fluorescent molecules were analysed by flow cytometry.

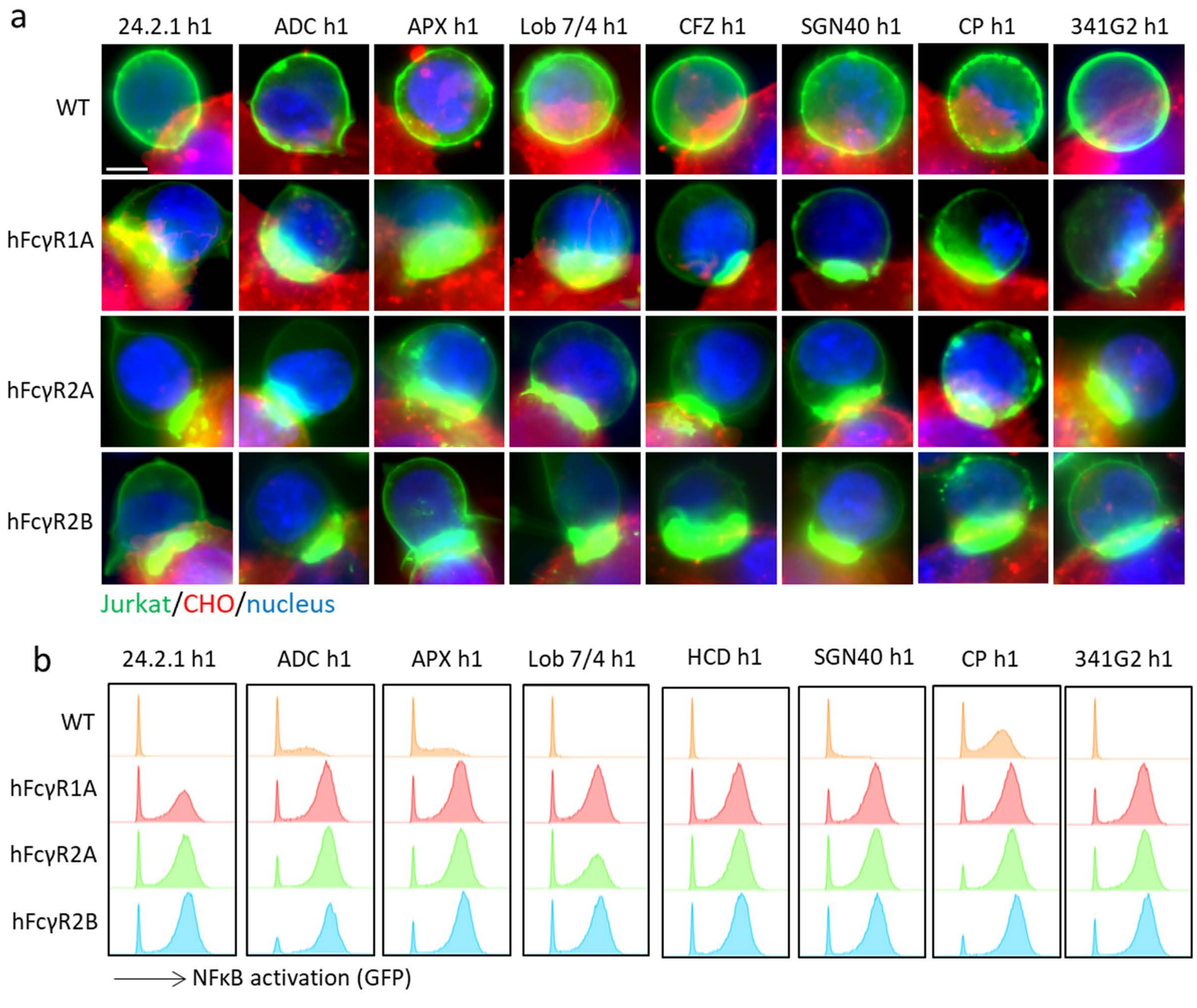
a
24.2.1 h1
ADC h1
APX h1
Lob 7/4 h1
CFZ h1
SGN40 h1
CP h1
341G2 h1
WT
hFcγR1A
hFcγR2A
hFcγR2B
Jurkat/CHO/nucleus
b
24.2.1 h1
ADC h1
APX h1
Lob 7/4 h1
HCD h1
SGN40 h1
CP h1
341G2 h1
WT
hFcγR1A
hFcγR2A
hFcγR2B
NFκB activation (GFP)

Supplementary Figure 3. FcγR-expressing CHO-k1 cells overcome anti-CD40 mAb epitope to mediate CD40 clustering. a Jurkat-hCD40ECD-GFP cells (green) opsonized with various anti-CD40 mAbs as indicated were incubated with CHO-k1 cells (red) expressing hFcγR1A, hFcγR2A or hFcγR2B for 15 minutes and then imaged in wide-field mode using an ONI Nanoimager; nuclei were labelled with Hoechst 33342 (blue). All images are representative of at least 10 images from 2 independent experiments. Scale bar, 4 µm. **b** Jurkat-NFκB-GFP reporter cells expressing hCD40 were incubated with various anti-CD40 mAbs as indicated at 10 µg/mL in the presence or absence of CHO-k1 cells expressing hFcγR1A, hFcγR2A or hFcγR2B for 6 hours, the level of NFκB activation was then quantified by GFP expression assessed by flow cytometry. Histograms representative of at least 3 independent experiments.

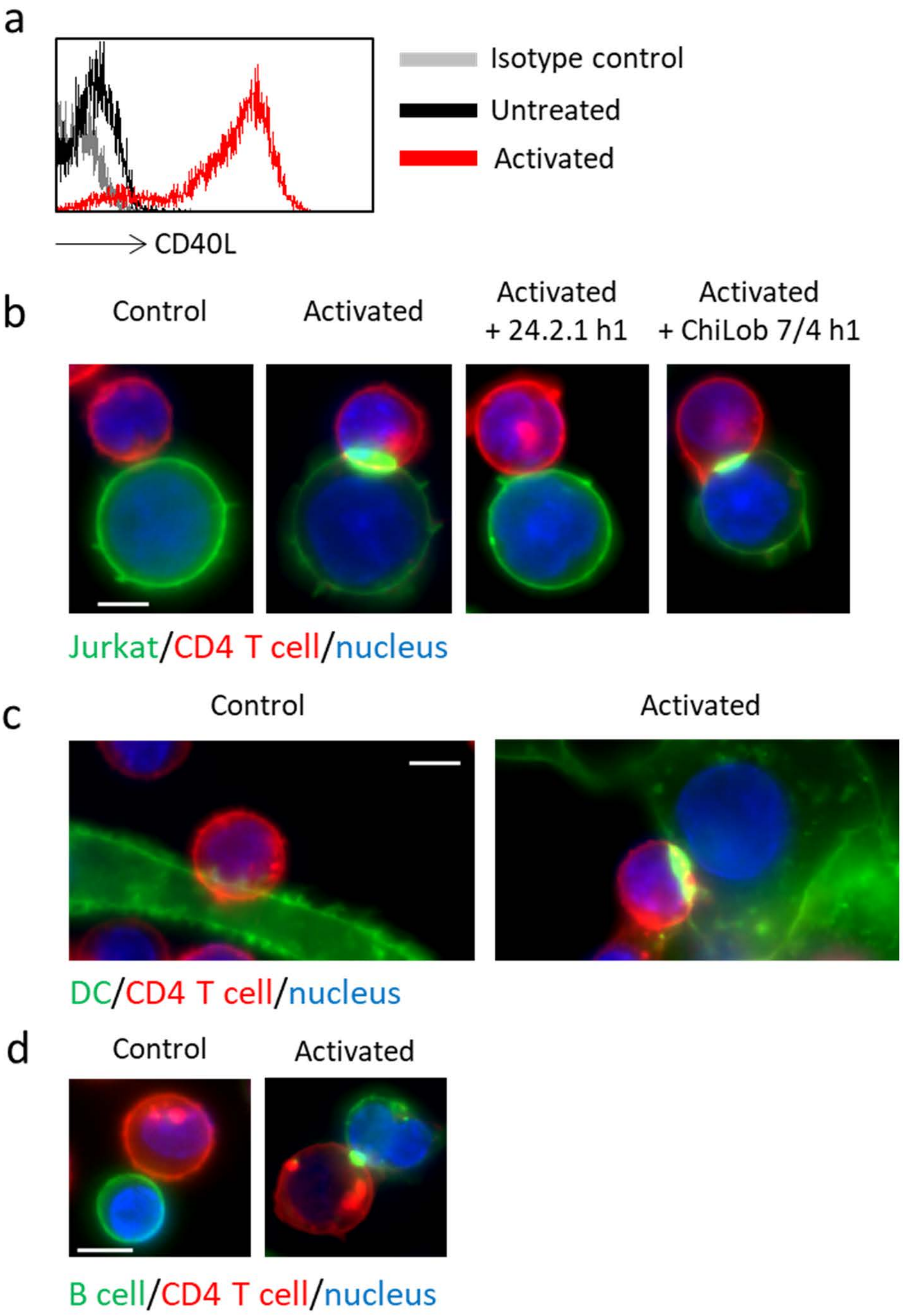

a
Isotype control
Untreated
Activated
CD40L
b
Control
Activated
Activated + 24.2.1 h1
Activated + ChiLob 7/4 h1
Jurkat/CD4 T cell/nucleus
c
Control
Activated
DC/CD4 T cell/nucleus
d
Control
Activated
B cell/CD4 T cell/nucleus

Supplementary Figure 4. Activated CD40L-expressing CD4 T cells induce CD40 clustering. a Purified human CD4 T cells were activated by incubation with PMA and ionomycin for 6 hours and then analysed for surface expression of CD40L by flow cytometry. **b-d** Activated T cells (red) were co-cultured with **b** Jurkat-hCD40ECD-GFP cells (green), **c** human monocyte-derived DCs pre-labelled with ChiLob 7/4 h1-AF488 (green), or **d** normal human B cells pre-labelled with ChiLob 7/4 h1-AF488 (green) for 20 minutes before wide-field fluorescence imaging using an ONI Nanoimager; nuclei were labelled with Hoechst 33342 (blue). All images are representative of at least 10 images from 2 independent experiments. Scale bar, 4 µm.

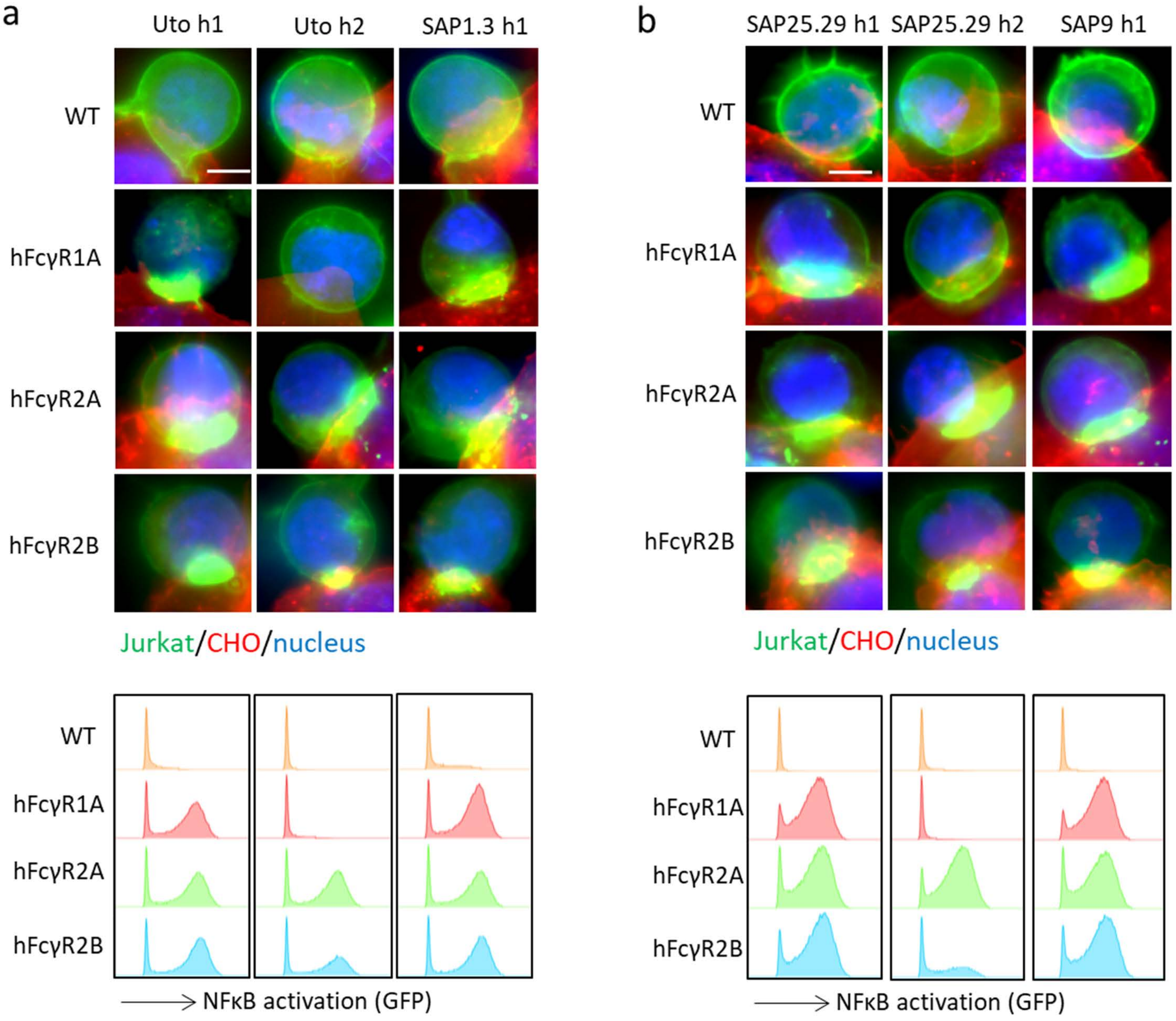
a
Uto h1
Uto h2
SAP1.3 h1
WT
hFcγR1A
hFcγR2A
hFcγR2B
Jurkat/CHO/nucleus
WT
hFcγR1A
hFcγR2A
hFcγR2B
NFκB activation (GFP)
b
SAP25.29 h1
SAP25.29 h2
SAP9 h1
WT
hFcγR1A
hFcγR2A
hFcγR2B
Jurkat/CHO/nucleus
WT
hFcγR1A
hFcγR2A
hFcγR2B
NFκB activation (GFP)

Supplementary Figure 5. FcγR-expressing CHO-k1 cells overcome anti-4-1BB mAb and anti-OX40 mAb epitope to mediate receptor clustering. a Jurkat-h4-1BBECD-GFP cells (green) opsonized with various anti-4-1BB mAbs as indicated were incubated with CHO-k1 cells (red) expressing hFcγR1A, hFcγR2A or hFcγR2B for 15 minutes and then imaged in wide-field mode using an ONI Nanoimager; nuclei were labelled with Hoechst 33342 (blue). Lower panels: Jurkat-NFκB-GFP reporter cells expressing h4-1BB were incubated with various anti-4-1BB mAbs as indicated at 10 μg/mL in the presence or absence of CHO-k1 cells expressing hFcγR1A, hFcγR2A or hFcγR2B for 6 hours. The level of NFκB activation was then quantified by GFP expression assessed by flow cytometry. **b** Jurkat-hOX40ECD-GFP cells (green) opsonized with various anti-OX40 mAbs as indicated were incubated with CHO-k1 cells (red) expressing hFcγR1A, hFcγR2A or hFcγR2B for 15 minutes and then imaged in wide-field mode using an ONI Nanoimager; nuclei were labelled with Hoechst 33342 (blue). Lower panels: Jurkat-NFκB-GFP reporter cells expressing hOX40 were incubated with various anti-OX40 mAbs as indicated at 10 μg/mL in the presence or absence of CHO-k1 cells expressing hFcγR1A, hFcγR2A or hFcγR2B for 6 hours. The level of NFκB activation was then quantified by GFP expression assessed by flow cytometry. All histograms are representative of at least 3 experiments; and all images are representative of at least 10 images from 2 independent experiments. Scale bar, 4 μm.

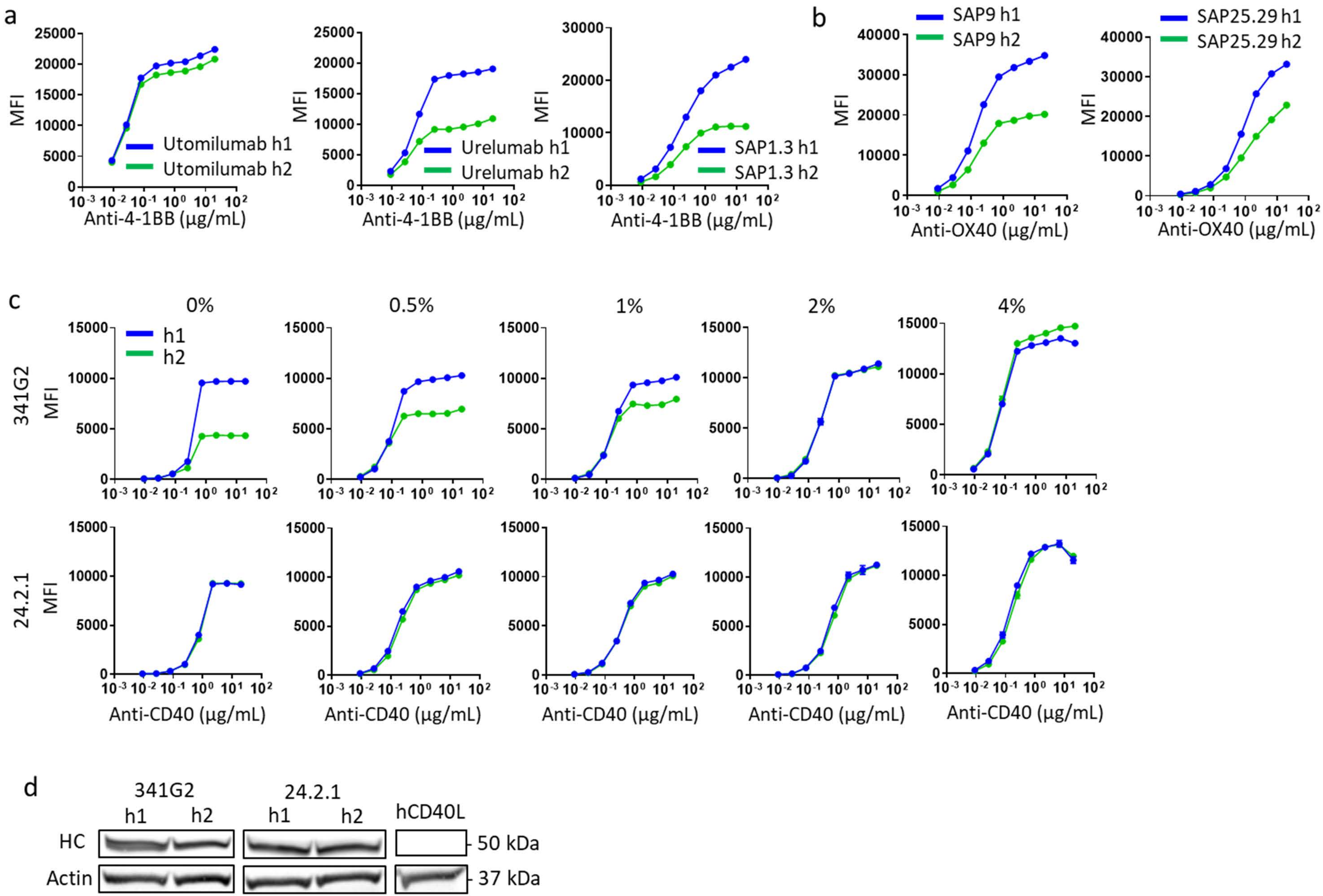
a
Utomilumab h1
Utomilumab h2
Urelumab h1
Urelumab h2
SAP1.3 h1
SAP1.3 h2
MFI
Anti-4-1BB (µg/mL)
b
SAP9 h1
SAP9 h2
SAP25.29 h1
SAP25.29 h2
Anti-OX40 (µg/mL)
c
0%
0.5%
1%
2%
4%
h1
h2
341G2
24.2.1
Anti-CD40 (µg/mL)
d
341G2
24.2.1
h1
h2
hCD40L
HC
Actin
50 kDa
37 kDa

Supplementary Figure 6. Receptor clustering causes epitope shielding. a Jurkat cells expressing h4-1BBECD were incubated with various anti-4-1BB mAbs as indicated for 1 hour and then washed and bound hIgG was detected by polyclonal anti-hIgG Fc. Means ± SEM, n = 3, data representative of independent 3 experiments. **b** Jurkat cells expressing hOX40ECD were incubated with various anti-OX40 mAbs as indicated for 1 hour and then washed and bound hIgG was detected by polyclonal anti-hIgG Fc. Means ± SEM, n = 3, data representative of 3 independent experiments. **c** Ramos cells were fixed with various concentrations of PFA as indicated and then washed and incubated with serially diluted 341G2 and 24.2.1 h1 or h2. Bound hIgG was detected by polyclonal anti-hIgG Fc. Means ± SEM, n = 3, data representative of 3 independent experiments. **d** Ramos cells were incubated with 10 µg/mL 341G2 and 24.2.1 h1 or h2 for 30 minutes and then cells were lysed and the lysates were subject to detection for hIgG by Western blotting as described in Methods. Data representative of 3 independent experiments.

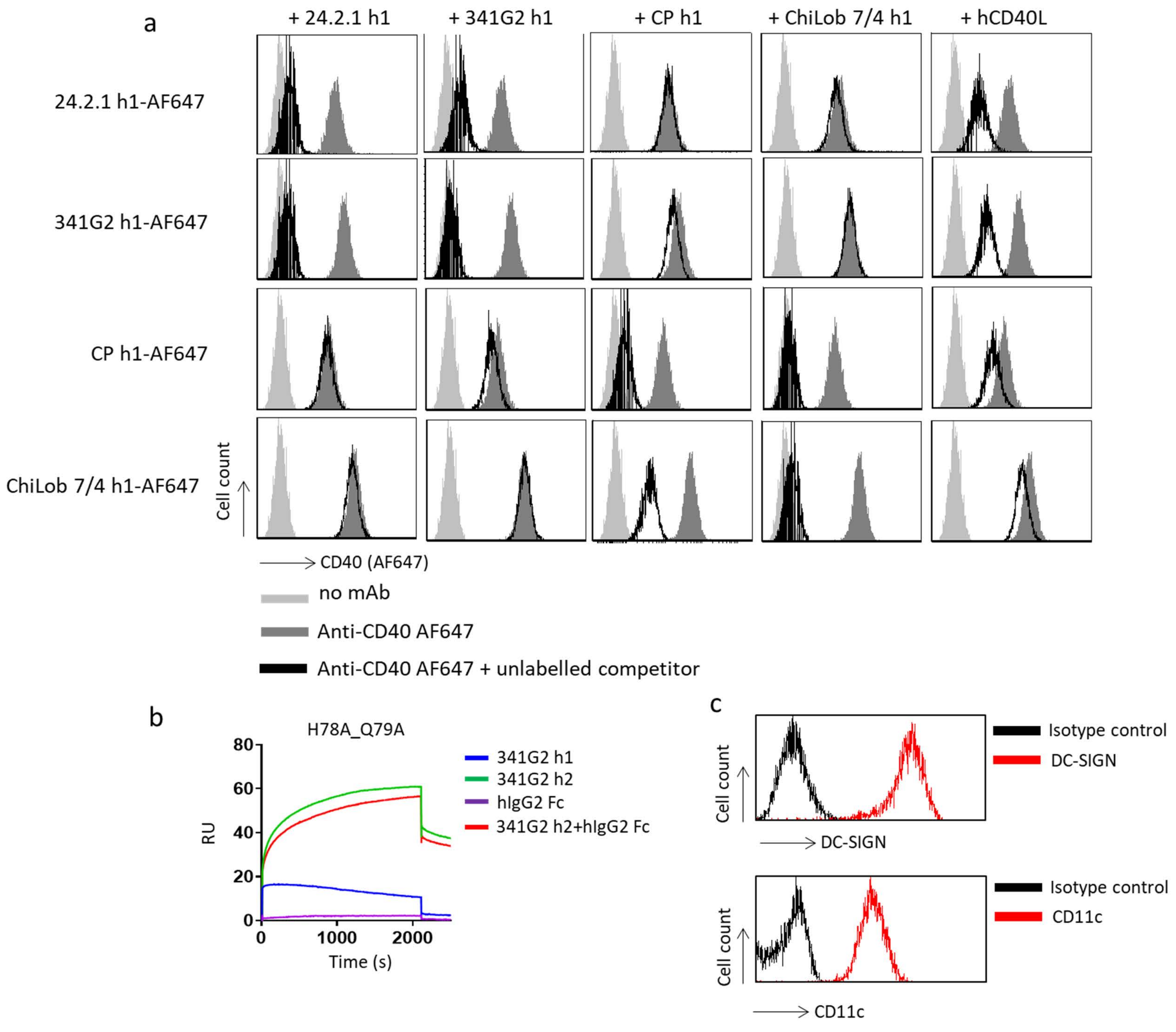

a
+ 24.2.1 h1
+ 341G2 h1
+ CP h1
+ ChiLob 7/4 h1
+ hCD40L
24.2.1 h1-AF647
341G2 h1-AF647
CP h1-AF647
ChiLob 7/4 h1-AF647
Cell count
CD40 (AF647)
no mAb
Anti-CD40 AF647
Anti-CD40 AF647 + unlabelled competitor
b
H78A_Q79A
RU
80
60
40
20
0
0
1000
2000
Time (s)
341G2 h1
341G2 h2
hIgG2 Fc
341G2 h2+hIgG2 Fc
c
Cell count
DC-SIGN
Isotype control
DC-SIGN
Cell count
CD11c
Isotype control
CD11c

Supplementary Figure 7. Anti-CD40 mAbs bind to different, non-cross-blocking, epitopes. a Ramos cells were incubated with 0.25 µg/mL AF647-labelled anti-CD40 mAb alone or in presence of 20 µg/mL of various unlabelled anti-CD40 mAbs as indicated, and the level of bound AF647-labelled mAb detected by flow cytometry. **b** Recombinant soluble His-tagged CD40ECD mutant was captured by anti-His mAb pre-immobilized onto a CM5 chip, and then 341G2 h1, 341G2 h2, recombinant hIgG2 Fc fragments or 341G2 h2 mixed with recombinant hIgG2 Fc fragments were injected at 1000 nM using a Biacore T200 instrument. The association phase lasted for 2100 seconds and the dissociation phase lasted for 300 seconds. **c** Immature DCs were derived from purified CD14+ monocytes cultured in the presence of IL-4 and GM-CSF for 6 days. The surface expression of DC-SIGN and CD11c on DCs was analysed by flow cytometry.

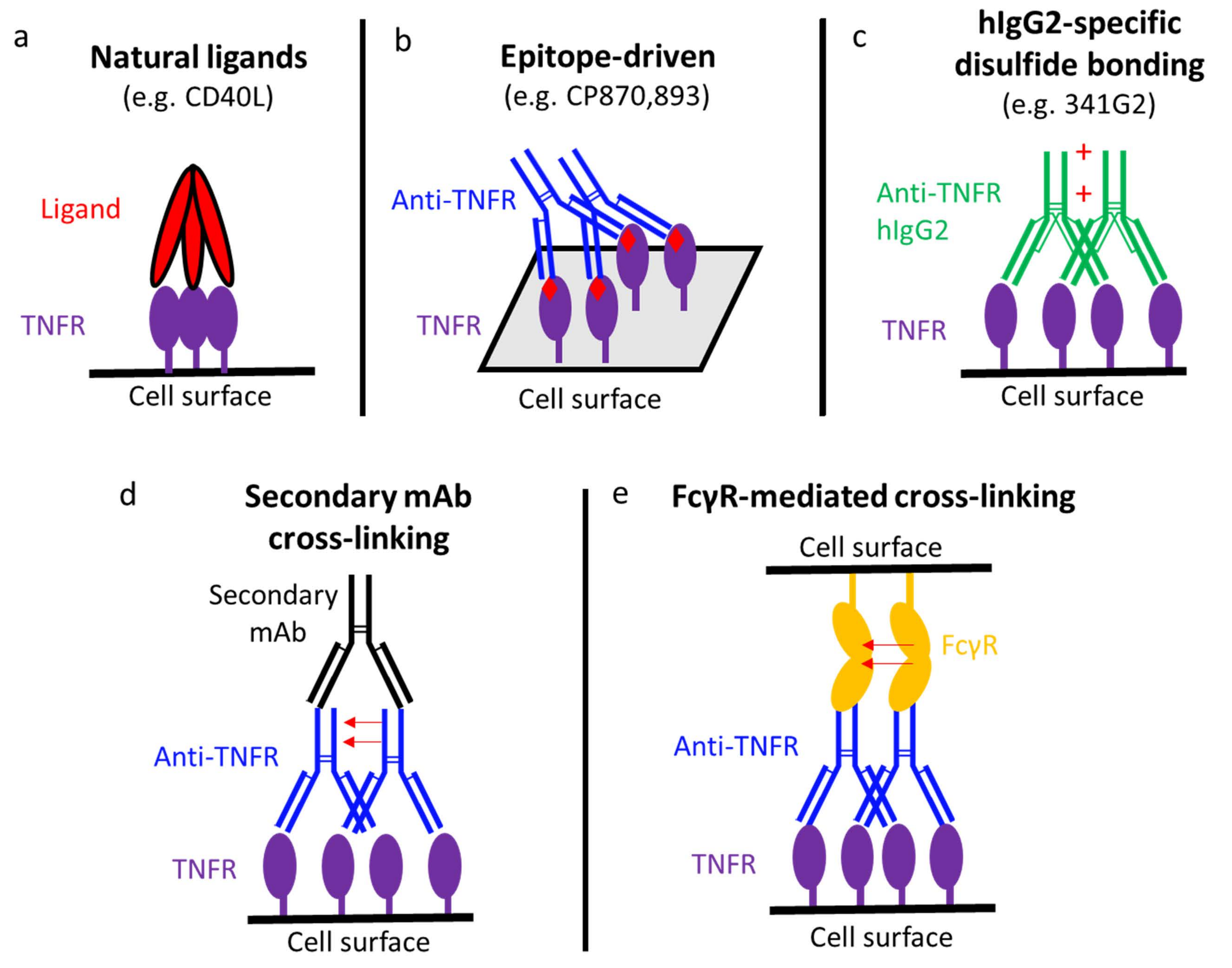
a
Natural ligands
(e.g. CD40L)
Ligand
TNFR
Cell surface
b
Epitope-driven
(e.g. CP870,893)
Anti-TNFR
TNFR
Cell surface
c
hIgG2-specific
disulfide bonding
(e.g. 341G2)
Anti-TNFR
hIgG2
TNFR
Cell surface
d
Secondary mAb
cross-linking
Secondary
mAb
Anti-TNFR
TNFR
Cell surface
e
FcγR-mediated cross-linking
Cell surface
FcγR
Anti-TNFR
TNFR
Cell surface

Supplementary Figure 8. Schematic indicating various means of achieving effective TNFR clustering. Natural ligands and TNFR-specific mAbs can achieve receptor clustering through a range of distinct modalities. **a** Natural ligands induce receptor clustering by virtue of their multimeric state, while antibodies can achieve receptor clustering through multiple alternative mechanisms: **b** by binding to rare epitopes that inherently facilitate clustering. **c** through effects mediated by the hIgG2 isotype. **d** through application of a second layer of hyper-crosslinking antibody. **e** through engagement with FcγR expressed on an accessory cell or surface.

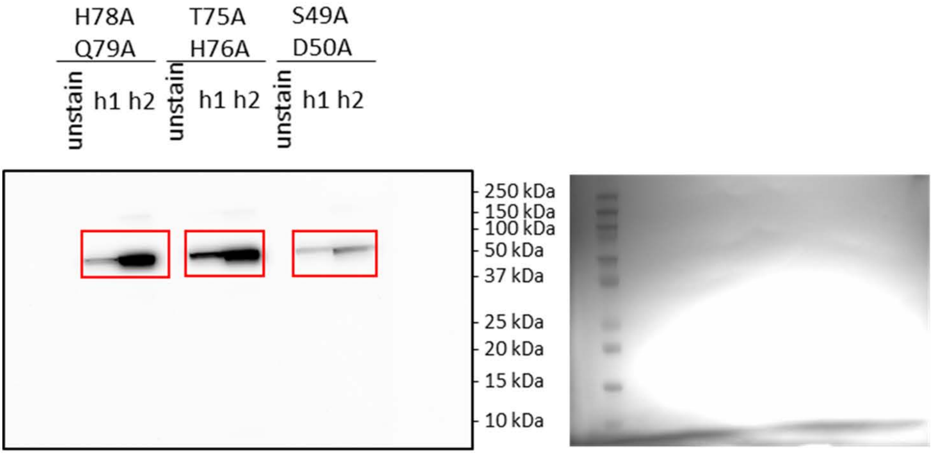
H78A
Q79A
T75A
H76A
S49A
D50A
unstain
h1 h2
unstain
h1 h2
unstain
h1 h2
250 kDa
150 kDa
100 kDa
50 kDa
37 kDa
25 kDa
20 kDa
15 kDa
10 kDa

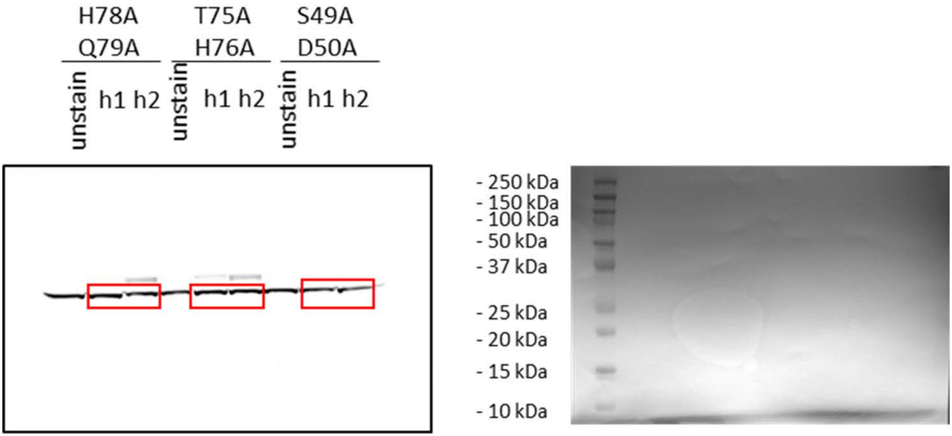
H78A
Q79A
T75A
H76A
S49A
D50A
unstain
h1 h2
unstain
h1 h2
unstain
h1 h2
250 kDa
150 kDa
100 kDa
50 kDa
37 kDa
25 kDa
20 kDa
15 kDa
10 kDa

Supplementary Figure 9. Full Western blotting images corresponding to Fig. 6d

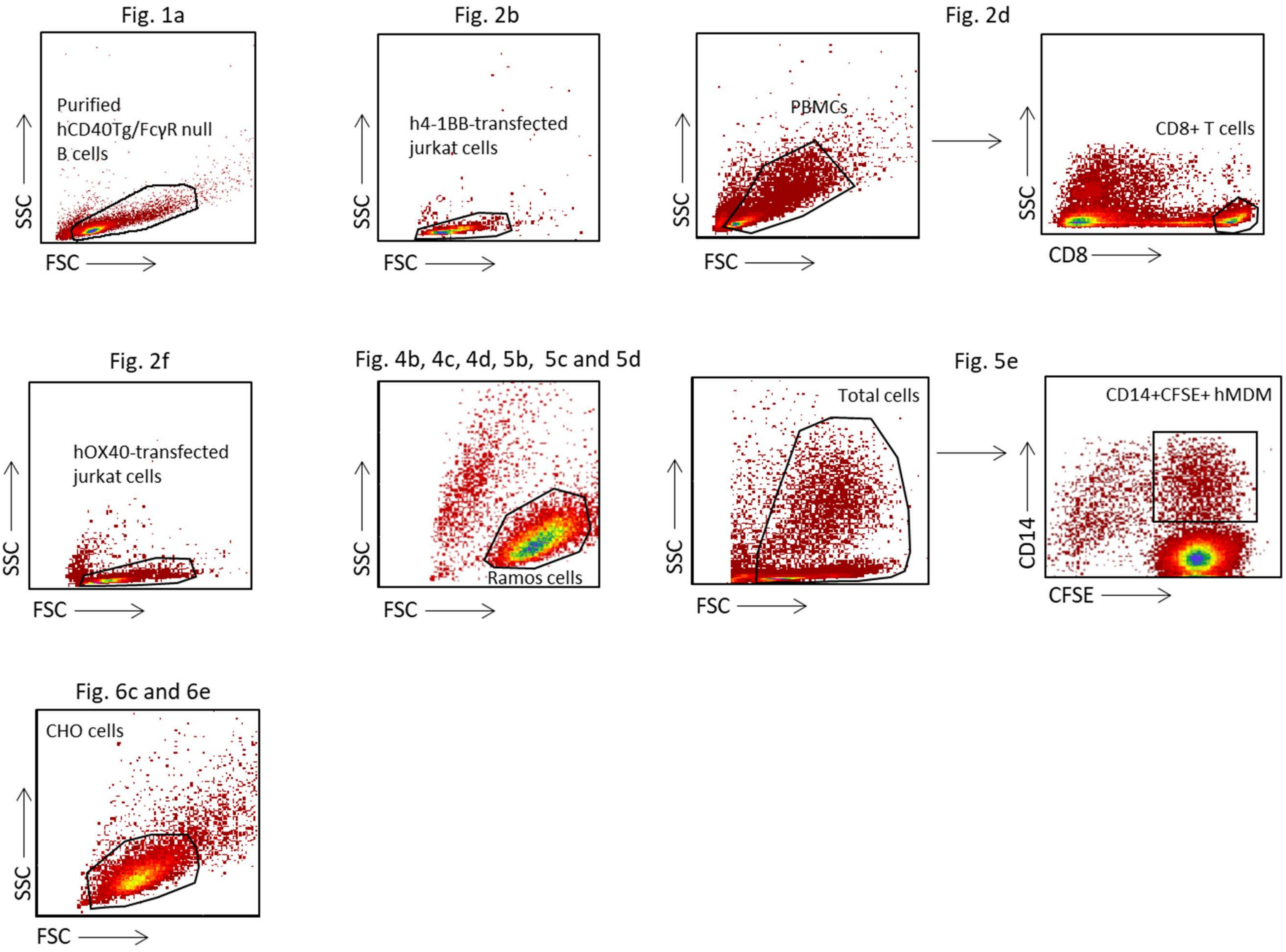

Fig. 1a
Purified hCD40Tg/FcγR null B cells
SSC
FSC
Fig. 2b
h4-1BB-transfected jurkat cells
SSC
FSC
Fig. 2d
PBMCs
SSC
FSC
CD8+ T cells
SSC
CD8
Fig. 2f
hOX40-transfected jurkat cells
SSC
FSC
Fig. 4b, 4c, 4d, 5b, 5c and 5d
Ramos cells
SSC
FSC
Fig. 5e
Total cells
SSC
FSC
CD14+CFSE+ hMDM
CD14
CFSE
Fig. 6c and 6e
CHO cells
SSC
FSC

Supplementary Figure 10. FACS gating strategy corresponding to Fig. 1a, 2b, 2d, 2f, 4b, 4c, 4d, 5b, 5c, 5d, 5e, 6c and 6e.

Supplementary References

1 Yu, X. *et al.* Isotype Switching Converts Anti-CD40 Antagonism to Agonism to Elicit Potent Antitumor Activity. *Cancer cell* **37**, 850-866 e857, doi:10.1016/j.ccell.2020.04.013 (2020).
