# Supplementary material for: TNF receptor agonists induce distinct receptor clusters to mediate differential agonistic activity
Source: Commun Biol. 2021 Jun 23;4:772. doi: 10.1038/s42003-021-02309-5 (PMC8222242; doi:10.1038/s42003-021-02309-5)
Supplement: Supplementary file 2 — Reporting Summary [file 42003_2021_2309_MOESM2_ESM.pdf]

nature research

Corresponding author(s): Mark Cragg
Last updated by author(s): May 25, 2021

Reporting Summary

Nature Research wishes to improve the reproducibility of the work that we publish. This form provides structure for consistency and transparency in reporting. For further information on Nature Research policies, see our Editorial Policies and the Editorial Policy Checklist.

Statistics

For all statistical analyses, confirm that the following items are present in the figure legend, table legend, main text, or Methods section.

n/a	Confirmed	
☐	☒	The exact sample size (*n*) for each experimental group/condition, given as a discrete number and unit of measurement
☐	☒	A statement on whether measurements were taken from distinct samples or whether the same sample was measured repeatedly
☐	☒	The statistical test(s) used AND whether they are one- or two-sided<br>*Only common tests should be described solely by name; describe more complex techniques in the Methods section.*
☒	☐	A description of all covariates tested
☐	☒	A description of any assumptions or corrections, such as tests of normality and adjustment for multiple comparisons
☐	☒	A full description of the statistical parameters including central tendency (e.g. means) or other basic estimates (e.g. regression coefficient) AND variation (e.g. standard deviation) or associated estimates of uncertainty (e.g. confidence intervals)
☐	☒	For null hypothesis testing, the test statistic (e.g. F, t, r) with confidence intervals, effect sizes, degrees of freedom and P value noted<br>*Give P values as exact values whenever suitable.*
☒	☐	For Bayesian analysis, information on the choice of priors and Markov chain Monte Carlo settings
☒	☐	For hierarchical and complex designs, identification of the appropriate level for tests and full reporting of outcomes
☒	☐	Estimates of effect sizes (e.g. Cohen's *d*, Pearson's *r*), indicating how they were calculated

Our web collection on statistics for biologists contains articles on many of the points above.

Software and code

Policy information about availability of computer code

Data collection	BD CellQuest and BD FACSDIVA was used to collect flow cytometry data<br>Leica Application Suite X was used to collect confocal microscopy data<br>STORM1.6 software from ONI UK was used to acquire STORM data<br>Visionworks 8.2 was used to control and collect data from UVP BioSpectrum Imaging System<br>BioTek Gen5 software was used to collect ELISA absorbances
Data analysis	NimOS v1.6 software from ONI UK was used to analyse STORM data<br>Leica Application Suite X was used to analyse confocal microscopy data<br>Biacore Bioevaluation software was used to analyse SPR data<br>ASTRA 6.1 (Wyatt Technologies) was used to analyse SEC-MALS data<br>GraphPad Prism was used for data analysis and to perform statistical tests<br>FlowJo was used to analyse flow cytometry data

For manuscripts utilizing custom algorithms or software that are central to the research but not yet described in published literature, software must be made available to editors and reviewers. We strongly encourage code deposition in a community repository (e.g. GitHub). See the Nature Research guidelines for submitting code & software for further information.

Data

Policy information about availability of data

All manuscripts must include a data availability statement. This statement should provide the following information, where applicable:

- Accession codes, unique identifiers, or web links for publicly available datasets
- A list of figures that have associated raw data
- A description of any restrictions on data availability

The datasets generated during and/or analyzed during the current study are available from the corresponding author on reasonable request.

Field-specific reporting

Please select the one below that is the best fit for your research. If you are not sure, read the appropriate sections before making your selection.

[x] Life sciences   [ ] Behavioural & social sciences   [ ] Ecological, evolutionary & environmental sciences

For a reference copy of the document with all sections, see nature.com/documents/nr-reporting-summary-flat.pdf

Life sciences study design

All studies must disclose on these points even when the disclosure is negative.

Sample size	For all in vitro experiments at least two independent experiments were performed to provides sufficient evidence for reproducibility. For human T cell proliferation assays at least 3 donors were used to provide sufficient evidence for reproducibility.
Data exclusions	No data was excluded from the analysis
Replication	In vitro experiments were repeated at least two independent times or with at least three independent donors.
Randomization	N/A
Blinding	N/A

Reporting for specific materials, systems and methods

We require information from authors about some types of materials, experimental systems and methods used in many studies. Here, indicate whether each material, system or method listed is relevant to your study. If you are not sure if a list item applies to your research, read the appropriate section before selecting a response.

Materials & experimental systems

n/a	Involved in the study	
[ ]	[x]	Antibodies
[ ]	[x]	Eukaryotic cell lines
[x]	[ ]	Palaeontology and archaeology
[ ]	[x]	Animals and other organisms
[x]	[ ]	Human research participants
[x]	[ ]	Clinical data
[x]	[ ]	Dual use research of concern

Methods

n/a	Involved in the study	
[x]	[ ]	ChIP-seq
[ ]	[x]	Flow cytometry
[x]	[ ]	MRI-based neuroimaging

Antibodies

Antibodies used

ChiLob 7/4, Lob 7/6, SAP1.3, SAP9 and SAP25.29 were generated in-house by conventional hybridoma method and isotype-switched to generated human IgG1 and IgG2. The variable domain sequences of 24.2.1 (US2009/0130715A1), 341G2 (US8716451B2), CP870,893 (US20090130715A1), ADC1013 (WO2016023960A1), APX005M (WO2014070934A1), SGN40 (WO2007075326A2), CFZ533 (WO2012075111A1), Urelumab (WO2010/042433A1), Utomilumab (WO2015/119923A1) and TGN1412 (US7585960B2) were derived from published patents and then their human IgG1 or IgG2 isotypes were generated in-house.
The following antibodies used in FACS were generated in house:
341G2 h1-mCherry, 341G2 h2-mCherry, 341G2 h1-AF647, 341G2 h2-AF647, 341G2 h1-AF488, 341G2 h2-AF488, Lob74 h1-AF647, CP h1-AF647, 24.2.1-AF647

The following antibodies were purchased from commercial suppliers:
PE anti-mouse CD23 (Biolegend, 101608)
PE anti-Human IgG (Abcam, ab98606)

	FITC monoclonal anti-human Fc (clone SB2H2, in-house)<br>AF647 anti-human Ig light chain κ (Biolegend, 316514)<br>rabbit monoclonal anti-human IgG (clone EPR4421, Abcam, ab109489)<br>rabbit anti-β-Actin (clone 13E5, Cell Signaling Technology, 4970S)<br>goat anti-rabbit IgG-HRP (Abcam, ab7090)<br>APC anti-human CD14 (Biolegend, 301808)<br>APC anti-human CD40L (Biolegend, 310810)<br>PE anti-human CD209 (DC-SIGN) (Biolegend, 330106)<br>PE-Cy5.5 anti-human CD11c (ebioscience, 15508856)<br>Pacific Blue anti-human CD8 (Biolegend, 344718)<br>APC anti-human CD8 (Biolegend, 344722)<br>Recombinant human IgG2 Fc (Sinobiological, 13504-HNAH)
Validation	The specificity of commercial antibodies was verified by the manufacturer, in-house antibodies were verified using cell lines specific to their species specificity and data in this manuscript support their specificity.

Eukaryotic cell lines

Policy information about cell lines

Cell line source(s)	Jurkat cells (ATCC), Ramos cells (ATCC), Jurkat and CHO cells stably transfected with human FcγR or CD40 were produced in-house
Authentication	Ramos cell line identity was confirmed using short tandem repeat analysis (Powerplex 16 System, Promega). CHO cells stably transfected with human FcγR were validated in-house using specific antibodies (Tutt et al JI 2015). Jurkat and CHO cells stably transfected with human CD40 were confirmed using anti-CD40 antibodies by flow cytometry.
Mycoplasma contamination	Mycoplasma test were conducted using the Mycoplasma: MycoAlert Mycoplasma Detection Kit (Lonza) and returned negative results.
Commonly misidentified lines<br>(See ICLAC register)	None were used in this study

Animals and other organisms

Policy information about studies involving animals; ARRIVE guidelines recommended for reporting animal research

Laboratory animals	hCD40Tg/FcγR null mice, aged 3-6 months
Wild animals	Study did not involve wild animals
Field-collected samples	Study did not involve samples collected from the field
Ethics oversight	All experiments were conducted under UK Home Office licence numbers PB24EEE31, P4D9C89EA, P540CBA98, and P39FE2AA7 and according to local ethical committee guidelines, reporting to the Home Office Animal Welfare Ethical Review Board (AWERB) at the University of Southampton.

Note that full information on the approval of the study protocol must also be provided in the manuscript.

Flow Cytometry

Plots

Confirm that:

- [x] The axis labels state the marker and fluorochrome used (e.g. CD4-FITC).
- [x] The axis scales are clearly visible. Include numbers along axes only for bottom left plot of group (a 'group' is an analysis of identical markers).
- [x] All plots are contour plots with outliers or pseudocolor plots.
- [x] A numerical value for number of cells or percentage (with statistics) is provided.

Methodology

Sample preparation	Cells from mouse spleens were harvested by dissociated using a cell strainer. All cells were prepared for flow cytometry by centrifugation for 5 minutes at 300g and resuspended in PBS supplemented with 1% BSA
Instrument	Flow cytometry was performed using FACS Calibur, FACS Canto II and LSR Fortessa instruments
Software	For FACS Calibur data were collected using BD Cell Quest and for FACS Canto II and LSR Fortessa data were collected using BD FACSDiva. Data was analysed using FlowJo

Cell population abundance	10,000 cells were collected for cell lines and 10,000 lymphocytes collected for human and mouse primary cell samples gated on their FSC/SCC properties.
Gating strategy	FSC/SCC gates were based on prior knowledge of the position of cells in a population based on these parameters due to the cells size and granularity. Positive cell populations were selected based on the observation of distinct populations that were stained for the cell marker being probed.

☒ Tick this box to confirm that a figure exemplifying the gating strategy is provided in the Supplementary Information.
